# Biomechanics of soft biological tissues and organs, mechanobiology, homeostasis and modelling

**DOI:** 10.1098/rsif.2024.0361

**Published:** 2025-01-29

**Authors:** Gerhard A. Holzapfel, Jay D. Humphrey, Ray W. Ogden

**Affiliations:** ^1^ Institute of Biomechanics, Graz University of Technology, Stremayrgasse, Austria; ^2^ Department of Structural Engineering, Norwegian University of Science and Technology, Trondheim, Norway; ^3^ Department of Biomedical Engineering and Vascular Biology & Therapeutics Program, Yale University and Yale School of Medicine, New Haven, CT, USA; ^4^ School of Mathematics and Statistics, University of Glasgow, Scotland, UK

**Keywords:** mechanobiology, biomechanics, soft biological tissue

## Abstract

The human body consists of many different soft biological tissues that exhibit diverse microstructures and functions and experience diverse loading conditions. Yet, under many conditions, the mechanical behaviour of these tissues can be described well with similar nonlinearly elastic or inelastic constitutive relations, both in health and some diseases. Such constitutive relations are essential for performing nonlinear stress analyses, which in turn are critical for understanding physiology, pathophysiology and even clinical interventions, including surgery. Indeed, most cells within load-bearing soft tissues are highly sensitive to their local mechanical environment, which can typically be quantified using methods of continuum mechanics only after the constitutive relations are determined from appropriate data, often multi-axial. In this review, we discuss some of the many experimental findings of the structure and the mechanical response, as well as constitutive formulations for 10 representative soft tissues or organs, and present basic concepts of mechanobiology to support continuum biomechanical studies. We conclude by encouraging similar research along these lines, but also the need for models that can describe and predict evolving tissue properties under many conditions, ranging from normal development to disease progression and wound healing. An important foundation for biomechanics and mechanobiology now exists and methods for collecting detailed multi-scale data continue to progress. There is, thus, considerable opportunity for continued advancement of mechanobiology and biomechanics.

## 1. Introduction

Soft biological tissues serve myriad functions, some mainly structural and some mainly functional. They consist of the same basic building blocks, including proteoglycans and collagen fibres, and yet exhibit a remarkable diversity in microstructure, biomechanical properties and overall geometry. Multiple imaging modalities, including brightfield, multi-photon, confocal and electron microscopy, provide exquisite detail of the microstructure of diverse tissues. Many different testing devices and methods, including standard uniaxial, biaxial and shear testing as well as new full-field optical methods, provide detailed information on the biomechanical behaviour *in vitro* while certain bioreactors can extend such information to *ex vivo* studies over short periods. Clinically available imaging, including computed tomography, magnetic resonance imaging and ultrasound, provides increasing detail on tissue and organ geometry *in vivo* and *in vitro*. Together, these many advances now provide unprecedented data at the tissue scale that can be modelled using methods of continuum biomechanics.

The continuum hypothesis proves convenient when characteristic length scales of the microstructure are much less than length scales of the physical problem, which holds for most biological tissues. For example, the diameter of a cell or collagen fibre (order of a few micrometres) is much less than that of the dimensions of most tissues and organs (often hundreds of micrometres and above). Not surprisingly then, continuum biomechanics has been highly successful in modelling the constitutive properties of tissues, and there are thus several motivations for further study. For example, (i) the primary function of many tissues and organs is mechanical, including the structural support provided by bones and the pumping function of the heart; (ii) cells, tissues and organs fail when mechanical stress exceeds mechanical strength, as e.g. when a bone breaks or an artery ruptures; and (iii) most cells within load-bearing tissues and organs are highly sensitive to changes in their local mechanical environment, often resulting in changes in gene expression and thus gene products. Associated adaptive changes include decreasing bone mass in microgravity environments or increasing skeletal muscle mass with exercise. Many more examples of mechano-regulated adaptive and maladaptive changes are identified easily. There is, therefore, significant motivation to study the allied fields of biomechanics and mechanobiology.

In this review, in §2, we summarize the requisite description of the structure and the mechanical behaviour of multiple healthy soft biological tissues, an essential starting point for understanding the normal mechanical environment experienced by cells as well as changes therein that result from disease progression and responses to injuries, including those surgically induced. Section 3 summarizes the related mechanobiology that underpins the discussion of micro-mechanics associated with cellular behaviour. In particular mechanical homeostasis, growth and remodelling in mechanobiology and recent advances in the mechanobiological modelling are discussed.

## 2. Biomechanics of some organs and soft biological tissues

### 2.1. Arteries

**Figure 1.**
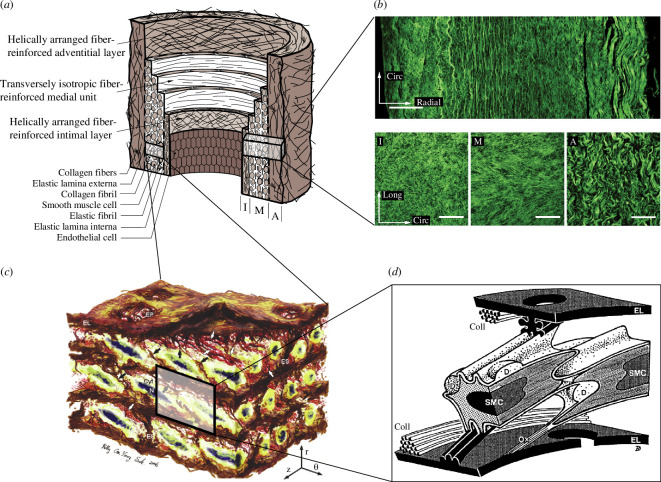
Structure of an artery: (*a*) healthy but aged aortic wall composed of three layers—intima (I), media (M) and adventitia (A); (*b*) healthy and aged abdominal aorta with intimal thickening indicating the layered collagen architecture. The top image shows the structure in the circumferential–radial plane and the bottom images show (I), (M) and (A) in the axial–circumferential plane (bars measure 100 μm); (*c*) detailed structure of an aortic media comprising several lamellar units, which include circumferentially oriented smooth muscle cells (SMCs) with elliptical nuclei shown in black between elastic lamellae (EL) and surrounded by a network of interlamellar elastic fibres; (*d*) schematic of the interconnections between two SMCs and two EL, and, in particular, collagen fibres (Coll). For a more detailed description see Dingemans *et al*. [1]. Adopted from Sherifova & Holzapfel [2].

The artery wall is composed of three main layers, the intima, media and adventitia. A schematic of an artery with intimal thickening is shown in figure 1*a*. The intima is not mechanically significant in a young and healthy artery and consists of a single layer of endothelial cells but it becomes mechanically significant with age [3].

The media comprises several concentric lamellar units each containing smooth muscle cells oriented relatively close to the circumferential direction, surrounded by collagen fibres and embedded in a proteoglycan-based interstitial matrix [4] (figure 1*c*,*d*). Collagen fibres are typically arranged in two families with their mean orientation closer to the circumferential direction than the axial direction. The media is the main load-bearing layer under physiological loading. In the adventitia there are also two symmetrically arranged families of collagen fibres with the mean orientation closer to the axial direction [5]. The adventitia acts as a stiff jacket-like tube at higher pressures, thus preventing overstretch and rupture of the artery [3]. The varying collagen fibre architectures through the layers of the wall are illustrated in figure 1*b* for a healthy abdominal aorta.

#### 2.1.1. Extension and distension of a thick-walled tube

From the modelling point of view, arteries can be considered as either a thin-walled or a thick-walled circular cylindrical tubes depending on the type of artery. Here, we briefly review the geometry and basic deformation of an artery undergoing extension and distension.

An intact, traction-free, thick-walled artery has geometry defined by e.g. section 5.3.3 in Ogden [6], i.e. A≤R≤B, 0≤Θ≤2π, 0≤Z≤L, where A,B,L are internal and external radii and length, respectively, and R,Θ,Z are cylindrical polar coordinates. The tube is deformed so that the circular cylindrical shape is maintained, and its current configuration is described in terms of cylindrical polar coordinates (r,θ,z). As the material is incompressible the deformation is given by


(2.1) $$r=f(R)\equiv [a^{2}+\lambda_{z}^{-1}(R^{2}-A^{2}){]}^{1/2},\;\theta =\Theta ,\;z=\lambda_{z}Z,$$

where $\lambda_{z}$ is the (uniform) axial stretch, a is the deformed internal radius and the function f is defined by (2.1)_1_.

The principal stretches $\lambda_{1},\lambda_{2},\lambda_{3}$, associated respectively with the radial, azimuthal and axial directions, are then


(2.2) $$\begin{array}{lcr} & \lambda_{1}=f^{\prime }(R)\;\lambda_{2}=\frac{r}{R}=\lambda_{\theta },\;\lambda_{3}=\lambda_{z}, & \end{array}$$

wherein the notation $\lambda_{\theta }$ is defined. By incompressibility $\lambda_{1}=\lambda_{2}^{-1}\lambda_{3}^{-1}=\lambda_{\theta }^{-1}\lambda_{z}^{-1}$.

Now consider two families of fibres embedded in the artery wall symmetrically arranged with respect to the axis of the artery, as e.g. in Schriefl *et al*. [5]. Let M and $M^{\prime }$ denote unit vectors which identify the fibre directions of the two families such that M has components cos⁡φ, sin⁡φ and $M^{\prime }$ has components cos⁡φ, −sin⁡φ with respect to the circumferential and axial directions, respectively, in terms of the angle φ.

Thus, the deformed configuration depends only on two stretches $\lambda_{\theta }$ and $\lambda_{z}$ and the angle φ. The elastic properties of such an artery are described by a strain-energy function $\Psi (\lambda_{\theta },\lambda_{z},\varphi )$.

With components of the Cauchy stress tensor denoted by $\sigma_{rr},\sigma_{\theta \theta }$ and $\sigma_{zz}$, we have e.g. Ogden [7]


(2.3) $$\begin{array}{lcr} & \lambda_{\theta }\frac{\partial \Psi }{\partial \lambda_{\theta }}=\sigma_{\theta \theta }-\sigma_{rr},\;\lambda_{z}\frac{\partial \Psi }{\partial \lambda_{z}}=\sigma_{zz}-\sigma_{rr}. & \end{array}$$

For the considered deformation, the only independent variable is r, and the local equilibrium equation reduces to the radial equation


(2.4) $$\begin{array}{lcr} & \frac{d\sigma_{rr}}{dr}+\frac{1}{r}(\sigma_{rr}-\sigma_{\theta \theta })=0. & \end{array}$$

Assuming that there is an internal pressure $p_{i}$ (on r=a) and no external load (on r=b), the boundary conditions are $\sigma_{rr}=-p_{i}$ and 0 on r=a and b, respectively. It followed by integration of (2.4) and use of (2.3)_1_ that


(2.5) $$\begin{array}{lcr} & p_{i}=\int_{a}^{b}\lambda_{\theta }\frac{\partial \Psi }{\partial \lambda_{\theta }}\frac{dr}{r}. & \end{array}$$

Noting from (2.1)_1_ with R=B that we have $b^{2}=a^{2}+\lambda_{z}^{-1}(B^{2}-A^{2})$, it is clear that (2.5) represents $p_{i}$ as a function of a for any given $\lambda_{z}$.

A fixed value of $\lambda_{z}$ during inflation requires an axial load on the ends of the tube. For a tube with closed ends, this includes a contribution $\pi a^{2}p_{i}$ from the pressure on the ends. When this contribution is removed, the remaining part of the load is that which must be applied to maintain $\lambda_{z}$ fixed. This is referred to as the reduced axial load, denoted F, and given by Holzapfel *et al*. [3], for example


(2.6) $$\begin{array}{lcr} & F=\pi \int_{a}^{b}(2\lambda_{z}\frac{\partial \Psi }{\partial \lambda_{z}}-\lambda_{\theta }\frac{\partial \Psi }{\partial \lambda_{\theta }})rdr. & \end{array}$$

As a particular example, we consider $\Psi =\Psi_{\mathrm{iso}}+\Psi_{\mathrm{fib}}$, where $\Psi_{\mathrm{iso}}$, equal to the neo-Hookean model $\Psi_{\mathrm{NH}}$, and $\Psi_{\mathrm{fib}}$ are given by


(2.7) $$\Psi_{NH}=\frac{1}{2}\mu (I_{1}-3),\;\Psi_{fib}=\frac{k_{1}}{k_{2}}\{\exp[k_{2}(I_{4}-1{)}^{2}]-1\},$$

where the invariants $I_{1}$ and $I_{4}$ are given by


(2.8) $$\begin{array}{lrr} & I_{1}=\lambda_{\theta }^{2}+\lambda_{z}^{2}+\lambda_{\theta }^{-2}\lambda_{z}^{-2},\;I_{4}=\lambda_{\theta }^{2}\cos^{2}\;\varphi +\lambda_{z}^{2}\sin^{2}\;\varphi . & \end{array}$$

**Figure 2.**
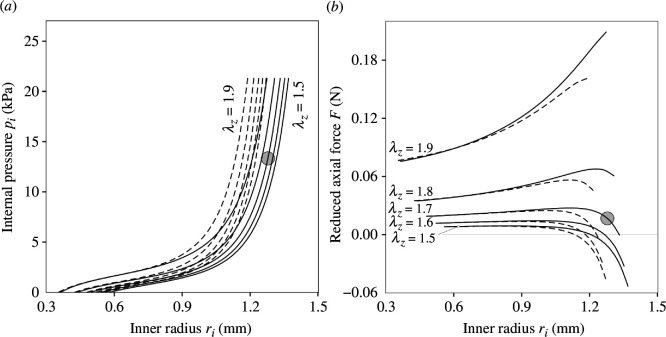
Plots of (*a*) internal pressure $p_{i}$ and (*b*) reduced axial force F versus the inner radius $r_{i}$ of a tube for several fixed values of the axial stretch $\lambda_{z}$ in respect to the constitutive model (2.7). The continuous curves refer to results with residual stresses and the dashed curves are results without residual stresses. The residual stresses were calculated based on an opening angle of $160.0^{\circ }$. The shaded circles indicate the approximate central region of the physiological state. Adopted from Holzapfel *et al*. [3].

These are then substituted in (2.5) and (2.6) to illustrate the dependence of the internal pressure $p_{i}$ and reduced axial force F on the inner radius $r_{i}=a$ of an artery. These formulae apply to multi-layered tubes with different properties for each layer. Calculations were carried out for a two-layered tube with residual stresses (relationships (2.1)_2_ and (2.2)_2_ are modified accordingly to take the opening angle into account) and fixed values of the axial stretch $\lambda_{z}$ included. Figure 2 shows plots of the dependence of $p_{i}$ and F on $r_{i}$ for several fixed values of the axial stretch with and without residual stresses. Geometrical data from Chuong & Fung [8] for a carotid artery from a rabbit (experiment 71 in Fung *et al*. [9]) were used with the assumption that the adventitia occupies one-third of the arterial wall thickness. For more details of the geometry including fibre orientation and identification of the material constants for each layer used, we refer to Holzapfel *et al*. [3]. The shaded circles identify the approximate location of the physiological state of this particular artery, which corresponds to $\lambda_{z}=1.7$ and a pressure of $p_{i}=13.33$ kPa. Note that the residual stress state in the arterial wall is three-dimensional and also layer-dependent. In particular, there are also residual stresses along the length of an artery, see e.g. the experimental study on human aortas with non-atherosclerotic intimal thickening [10], with the related model documented in Holzapfel & Ogden [11], and an extension to consider an anisotropic function for the prediction of residual stresses in Sigaeva *et al*. [12]. Additional constitutive relations and motions (including torsion, important for the ascending aorta) for arteries and veins can be found in Humphrey [13].

### 2.2. Myocardium

**Figure 3.**
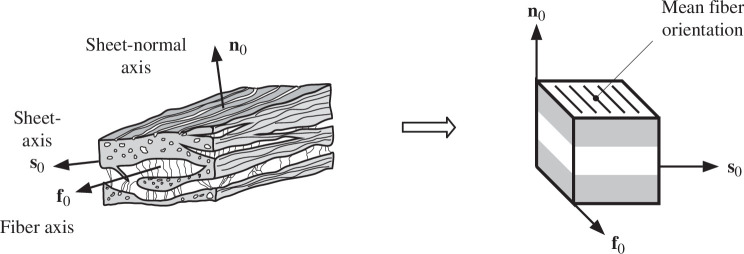
Layered organization of the sheets of myocytes with collagen fibres between the sheets referred to the orthonormal fibre axis ${\text{f}}_{0}$, sheet axis ${\text{s}}_{0}$ and sheet-normal axis ${\text{n}}_{0}$, mapped into a tissue cube with the three axes. Adopted from Holzapfel & Ogden [15].

Like arteries, the wall of the heart consists of three layers: endocardium (inner), myocardium (middle) and epicardium (outer). Whereas the endocardium and epicardium are thin membrane-like tissues consisting primarily of collagen, the myocardium represents the bulk of the wall. The myocardium has a layered structure consisting of myocytes connected by endomysial collagen surrounding and connecting adjacent myocytes. The muscles are arranged in layers which are three–four cells thick. The layered structure is represented by a set of orthonormal basis vectors with corresponding curvilinear coordinates. The direction of the myocytes in an appropriate reference configuration is identified by the fibre axis denoted ${\text{f}}_{0}$. The sheet axis, denoted ${\text{s}}_{0}$, lies in the layer plane normal to the fibre direction, while ${\text{n}}_{0}$, the sheet-normal axis, completes the right-handed set of basis vectors [14]. The structure is presented schematically in figure 3 with the cube and local basis vectors. This provides a basis for a geometrical and constitutive model of the myocardium, in which the labels f, s and n refer to fibre, sheet and normal, respectively, while the pairs fs, fn and sn refer to the fibre-sheet, fibre-normal and sheet-normal planes.

#### 2.2.1. A specific material model

In order to represent data from simple shear experiments, we introduce the specific energy function


(2.9) $$\Psi =\frac{a}{2b}\exp[b(I_{1}-3)]+\sum_{i=f,s}\frac{a_{i}}{2b_{i}}\left\{\exp[b_{i}(I_{4\;i}-1{)}^{2}]-1\right\}+\frac{a_{fs}}{2b_{fs}}\left[\exp(b_{fs}I_{8\;fs}^{2})-1\right],$$

given in Holzapfel & Ogden [15], from which the Cauchy stress tensor is calculated as


(2.10) $$\begin{array}{rcl} \sigma & = & -p\text{I}+a\exp[b(I_{1}-3)]\text{b}+2a_{f}(I_{4\;f}-1)\exp[b_{f}(I_{4\;f}-1{)}^{2}]\text{f}⊗\text{f}\\ & & +\;2a_{s}(I_{4\;s}-1)\exp[b_{s}(I_{4\;s}-1{)}^{2}]\text{s}⊗\text{s}+a_{fs}I_{8\;fs}\exp(b_{fs}I_{8\;fs}^{2})(\text{f}⊗\text{s}+\text{s}⊗\text{f}), \end{array}$$

where $a,a_{f},a_{s},a_{\mathrm{fs}}$ (kPa), $b,b_{f},b_{s},b_{\mathrm{fs}}$ (dimensionless) are positive material constants, with the three invariants $I_{4\;f}={\text{f}}_{0}\cdot (\text{C}{\text{f}}_{0})$, $I_{4\;s}={\text{s}}_{0}\cdot (\text{C}{\text{s}}_{0})$ and $I_{8\;fs}={\text{f}}_{0}\cdot (\text{C}{\text{s}}_{0})$. The $I_{1}$-related term provides an isotropic contribution, while the terms in $I_{4\;f}$ and $I_{4\;s}$ are transversely isotropic in form and the term in $I_{8\;\mathrm{fs}}$ is orthotropic.

As a simple example, we analyse simple shear in the fs plane by considering shear in the ${\text{f}}_{0}$ and ${\text{s}}_{0}$ directions. The corresponding deformation gradients have component forms


(2.11) $$[\text{F}]=\left[\begin{array}{ccc} 1 & \gamma & 0\\ 0 & 1 & 0\\ 0 & 0 & 1 \end{array}\right],\;[\text{F}]=\left[\begin{array}{ccc} 1 & 0 & 0\\ \gamma & 1 & 0\\ 0 & 0 & 1 \end{array}\right],$$

where γ is the amount of shear. For shear in the ${\text{f}}_{0}$ direction, we have $\text{f}={\text{f}}_{0}$, $\text{s}=\gamma {\text{f}}_{0}+{\text{s}}_{0}$ and $\text{n}={\text{n}}_{0}$, with $I_{4\;s}=1+\gamma^{2}$, $I_{4\;f}=1$, while for shear in the ${\text{s}}_{0}$ direction we have $\text{f}={\text{f}}_{0}+\gamma {\text{s}}_{0}$, $\text{s}={\text{s}}_{0}$, $\text{n}={\text{n}}_{0}$, with $I_{4\;f}=1+\gamma^{2},I_{4\;s}=1$. The results are similar for shear in the fn and sn planes. For details, we refer to Holzapfel & Ogden [16].

**Figure 4.**
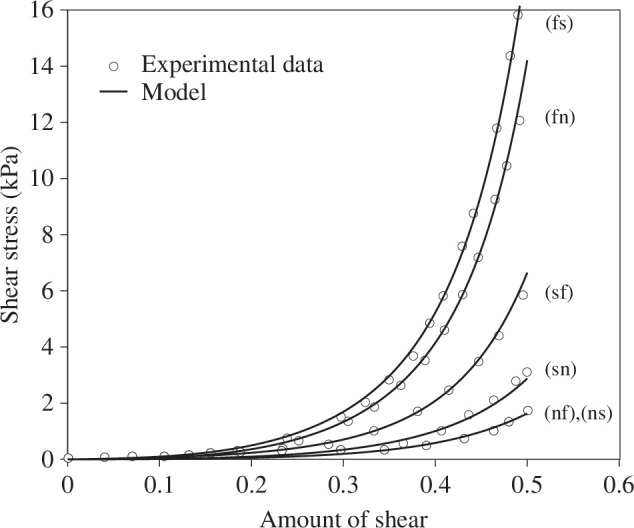
Fit of the shear components of the Cauchy stress (2.10) to the experimental data for the loading curves. Adopted from Dokos *et al*. [17].

The model is used to fit data from the six distinct shear modes reported by Dokos *et al*. [17], with figure 4 showing the fit of the model to the data. Note here that the nf and ns shear curves are indistinguishable for the considered set of data. The material parameters are a=0.059 kPa, b=8.023, $a_{f}=18.472$ kPa, $b_{f}=16.026$, $a_{s}=2.481$ kPa, $b_{s}=11.120$, $a_{\mathrm{fs}}=0.216$ kPa and $b_{\mathrm{fs}}=11.436$. Note, in particular, the difference in the two shear stresses in each plane.

#### 2.2.2. Representative numerical illustration

Myocardium behaves as a viscoelastic material according to the data documented by Sommer *et al*. [18]. Thus, the incorporation of viscoelastic effects can improve the constitutive model. Based on the framework of viscoelasticity described by Nordsletten *et al*. [19], consider an application to an idealized left ventricle geometry. In particular, we compare viscoelastic results with those for the purely elastic model and discuss the pressure–volume relationship during one cardiac cycle for the left atrium and the left ventricle. The left ventricle is modelled with an ellipsoidal geometry and a rule-based distribution of collagen fibres, with myocardial activation applied to generate a more realistic contraction pattern. Full details are in Zhang *et al*. [20].

**Figure 5.**
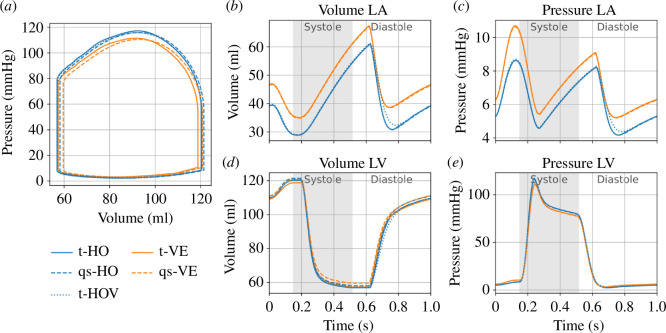
(*a*) Pressure–volume loop for the left ventricle. (*b*,*c*) Volume–pressure traces for the cardiac cycle for the left atrium (LA). (*d*,*e*) Volume–pressure traces for the cardiac cycle for the left ventricle (LV). Blue (orange) curves are based on the HO models (VE models). Solid curves indicate the use of transient mechanics, while dashed curves show the quasi-static approach. Dotted curves indicate solutions where an artificial dampening was added to the transient HO simulation. Adapted from Zhang *et al*. [20].

An example is shown in figure 5. The abbreviation HO refers to the model in Holzapfel & Ogden [15] (§2.2.1 of the current article), while VE stands for viscoelasticity and refers to the model in Nordsletten *et al*. [19]. The prefix t refers to transient and prefix qs refers to quasi-static, while t-HOv refers to the transient solution of the HO model with artificial viscosity included. In figure 5*a*, the pressure–volume loop for the left ventricle is shown for each of the models. By comparison with the HO models, VE models show a slight decrease in stroke volume due to a decrease in end-diastolic volume and an increase in end-systolic volume. This can also be seen in the volume trace in figure 5*d*. For more detailed discussion of the slight differences in the results for the left ventricle, we refer to section 6.2 in Zhang *et al*. [20]. The main differences between the results of the VE and the HO models occur in the left atrium where the VE models exhibit higher pressures and volumes, see figure 5*b*,*c*.

An integrative simulator for human heart function within the framework of the Living Heart Project was proposed by Baillargeon *et al*. [21]. This prototype model consists of a four-chamber human heart generated from imaging. It allows a better understanding of the key features, physics and technologies to create a predictive model incorporating solid and fluid mechanics and electrophysiology. Recent developments include the coupling of a three-dimensional model of bi-ventricular electro-mechanics to a physiological model representing atrial mechanics with closed-loop circulation [22]. This type of model has the potential to predict the outcomes of electro-mechanical therapies. Another comprehensive model integrates cardiac electrophysiology, active and passive mechanics and haemodynamics [23], and it includes reduced-order models for cardiac valves and the circulatory system. The model is capable of representing the period of the heartbeat and its phases, in accordance with clinical data. It also correctly represents iso-volumetric phases of the heartbeat and reproduces the effects associated with travelling pressure waves.

### 2.3. Brain tissue

Soft tissues of the brain have a complex structure and are ultra-soft. Recently, it was shown that mechanics plays a key role in both neural function and dysfunction [24,25]. Suitable mechanical models are required to capture the highly adaptive and heterogeneous properties of the tissue to provide realistic predictions of mechanobiological processes in the brain. From the macroscopic point of view, the grey and white matter tissue can be considered separately. Grey matter consists mostly of neurons that are involved in data processing, while white matter consists of nerve fibres that facilitate a rapid transduction of signals. Even within these two types of tissues the microstructures vary significantly. Detailed testing of the mechanical properties of the tissues has been carried out by Franceschini *et al*. [26], Haslach *et al*. [27] and Budday *et al*. [28], where it has been shown that brain tissue is both nonlinear and poroviscoelastic, and can be well represented with an isotropic model. A detailed description of the brain tissue and its mechanical properties including modelling aspects can be found in the extensive review by Budday *et al*. [29].

#### 2.3.1. A specific material model

We now consider a specific form of a model suitable for representing data on brain tissue. It has been shown by Budday *et al*. [29] that a range of experimental data for brain tissue can be described by a single-term isotropic model of the Ogden-type. Here, we therefore consider separately the equilibrium part ${\bar{\Psi }}_{Oeq}$ and the non-equilibrium part ${\bar{\Psi }}_{Oneq}$ which are added to provide the total energy function based on the more general three-term Ogden model [30].

First, the isochoric equilibrium part ${\bar{\Psi }}_{Oeq}$ can be expressed as


(2.12) $${\bar{\Psi }}_{Oeq}=\sum_{i=1}^{3}\frac{\mu_{i}}{\alpha_{i}}({\bar{\lambda }}_{1}^{\alpha_{i}}+{\bar{\lambda }}_{2}^{\alpha_{i}}+{\bar{\lambda }}_{3}^{\alpha_{i}}-3),$$

where $\mu_{i}$ and $\alpha_{i}$ are equilibrium material parameters, while ${\bar{\lambda }}_{i}$, i=1,2,3, are isochoric principal stretches defined by ${\bar{\lambda }}_{i}=J^{-1/3}\lambda_{i}$, where $J=\lambda_{1}\lambda_{2}\lambda_{3}$.

Second, the isochoric non-equilibrium part of the free-energy function, denoted by ${\bar{\Psi }}_{Oneq}$, is isochoric as well and expressed in terms of the isochoric elastic principal stretches ${\bar{\lambda }}_{i}^{e}=(J^{e}{)}^{-1/3}\lambda_{i}^{e}$, i=1,2,3, as


(2.13) $${\bar{\Psi }}_{Oneq}=\sum_{i=1}^{3}\frac{\nu_{i}}{\beta_{i}}\left[({\bar{\lambda }}_{1}^{e}{)}^{\beta_{i}}+({\bar{\lambda }}_{2}^{e}{)}^{\beta_{i}}+({\bar{\lambda }}_{3}^{e}{)}^{\beta_{i}}-3\right],$$

where $\nu_{i}$ and $\beta_{i}$ are non-equilibrium material parameters. Note that we used the multiplication decomposition of the principal stretches $\lambda_{i}=\lambda_{i}^{e}\lambda_{i}^{v}$, where $\lambda_{i}^{e}$ denote the elastic principal stretches and $\lambda_{i}^{v}$ the related viscoelastic principal stretches in addition to the elastic part of the volume ratio $J^{e}=\lambda_{1}^{e}\lambda_{2}^{e}\lambda_{3}^{e}$.

The total Kirchhoff stress tensor τ is separated into isochoric, viscoelastic and volumetric parts so that


(2.14) $$\tau =\tau_{Oeq}+\tau_{Oneq}+\tau_{vol},$$

where $\tau_{Oeq}$ is


(2.15) $$\tau_{Oeq}=\sum_{i,j=1}^{3}\mu_{i}\left[{\bar{\lambda }}_{j}^{\alpha_{i}}-\frac{1}{3}({\bar{\lambda }}_{1}^{\alpha_{i}}+{\bar{\lambda }}_{2}^{\alpha_{i}}+{\bar{\lambda }}_{3}^{\alpha_{i}})\right]{\text{n}}_{j}⊗{\text{n}}_{j},$$

and $\tau_{Oneq}$ is


(2.16) $$\tau_{Oneq}=\sum_{i,j=1}^{3}\nu_{i}\left[\left({\bar{\lambda }}_{j}^{e}{)}^{\beta_{i}}-\frac{1}{3}[({\bar{\lambda }}_{1}^{e}{)}^{\beta_{i}}+({\bar{\lambda }}_{2}^{e}{)}^{\beta_{i}}+({\bar{\lambda }}_{3}^{e}{)}^{\beta_{i}}\right]\right]{\text{n}}_{j}^{e}⊗{\text{n}}_{j}^{e},$$

where ${\text{n}}_{j}$ are the eigenvectors of the left Cauchy–Green tensor $\text{b}=\sum_{i=1}^{3}\lambda_{i}^{2}{\text{n}}_{i}⊗{\text{n}}_{i}$, and ${\text{n}}_{j}^{e}$ are the three eigenvectors of the elastic part of the left Cauchy–Green tensor ${\text{b}}^{e}=\sum_{i=1}^{3}(\lambda_{i}^{e}{)}^{2}{\text{n}}_{i}^{e}⊗{\text{n}}_{i}^{e}$. In addition, $\tau_{vol}$ is the volumetric contribution defined by $JU^{\prime }(J)\text{I}$, where U(J) denotes a volumetric function and the prime denotes the derivative with respect to J. For the derivation of the isochoric Kirchhoff stresses, we refer to Holzapfel [31].

To *a priori* ensure non-negative dissipation, assuming isotropy, a possible choice of an evolution equation for the internal variable ${\text{b}}^{e}$ is [32]


(2.17) $$-{£}_{v}({\text{b}}^{e})({\text{b}}^{e}{)}^{-1}=\frac{1}{\eta }\tau_{Oneq},$$

where the introduced parameter η>0 is the viscosity and ${£}_{v}(∙)$ denotes the Lie derivative of (∙) in the direction of v [31]. It can be shown that this choice leads to a dissipation inequality of the quadratic form, i.e. $(\tau_{Oneq}:\tau_{Oneq})/(2\eta )\geq 0$, which is necessarily non-negative.

#### 2.3.2. Comparison with experimental results

**Figure 6.**
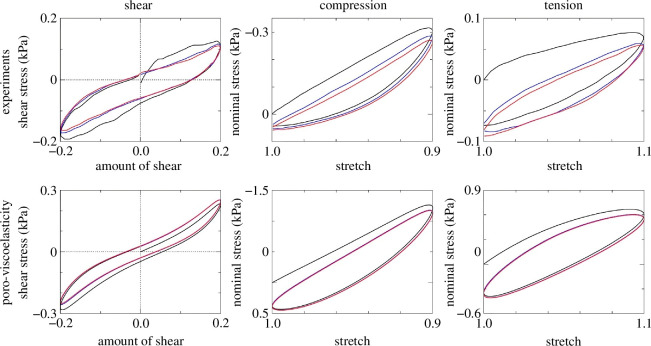
Shear, compressive and tensile tests of human brain tissue from Budday *et al*. [35] (top row) showing three consecutive loading cycles (first cycle is coloured black, the second and third coloured blue and red respectively). Numerical results from a poroviscoelastic model (bottom row) indicate a good agreement with experimental data. Modified from Comellas *et al*. [34].

The specific finite viscoelastic model as outlined in §2.3.1 was combined with nonlinear poroelasticity following the theory of porous media and implemented in a finite element (FE) framework using the open-source FE library deal.II [33]. For details of the time discretization of the governing equations and the FE implementation, see Comellas *et al*. [34]. In particular, shear, compressive and tensile tests on cubes with the dimensions 5×5×5 mm were simulated with a total of 512 elements. Detailed boundary and loading conditions are described in fig. 6 of Comellas *et al*. [34]. Figure 6 here shows numerical results of the poroviscoelastic model for the three different deformation modes in comparison with experimental results documented by Budday *et al*. [35]. The stress–deformation behaviour indicates a strong hysteresis during three consecutive cycles of loading; the first cycle is coloured black, the second cycle is blue and the third cycle is red. It is important to note that nonlinear viscoelasticity alone is not able to capture the experimental data, as also mentioned by Comellas *et al*. [34].

### 2.4. Articular cartilage

**Figure 7.**
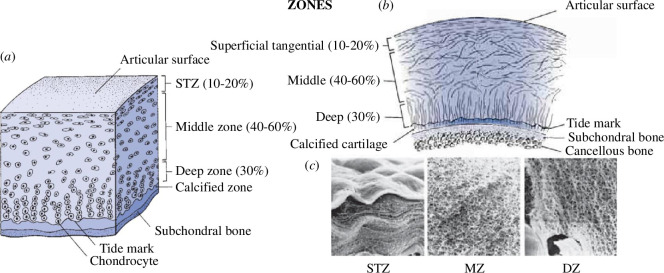
(*a*) Schematic of a healthy articular cartilage consisting of three zones: a superficial tangential zone (STZ), middle zone (MZ) and deep zone (DZ). There is also a non-homogeneous distribution of chondrocytes, which is denser towards the articular surface and the cancellous bone. (*b*) Illustration of the collagen fibre architecture for each individual zone (orientation for (*a*) and (*b*) is for gravity acting downwards). (*c*) Examples of photomicrographs (×3000) of the arrangement of the collagen network through the thickness of the articular cartilage. Modified from Mow & Lai [36] and Mow & Hung [37].

Articular cartilage is composed of a small number of chondrocytes surrounded by a multi-component matrix; for the particular cell organization through the thickness of the articular cartilage see figure 7*a*. The chondrocytes are sparsely distributed except near the articular surface and the cancellous bone. Near the articular surface the chondrocytes are elongated and aligned along the surface, while towards the bone they are arranged in columns perpendicular to the bone. However, in the middle of the cartilage, the chondrocytes are circular in shape and randomly distributed. Remarkably, 70–85% of the tissue weight is water and the rest of the tissue is mainly composed of collagen and proteoglycans, with the proteoglycan concentration and water content varying through the thickness.

Nearest to the articular surface is a layer which is between 10% and 20% of the tissue thickness; this is called the superficial tangential zone (STZ) with the collagen oriented essentially tangentially to the surface and arranged in tightly woven sheets. The middle zone (MZ), consists of 40–60% of the thickness, with a collagen fibre distribution considered to be isotropic and less dense than in the STZ. The deep zone (DZ), has a thickness of about 30% of the tissue with the collagen fibres oriented essentially orthogonal to the surface of the bone, and arranged in tightly packed bundles, as depicted in figure 7*b*, and anchored to the underlying bone. Both MZ and DZ are responsible for bearing compressive load. The thickness of the collagen fibres is least in the STZ and is greatest in the DZ. Note that the arrangement and shape of the chondrocytes is consistent with the collagen fibre structure. Figure 7*c* shows photomicrographs of the collagen arrangements in the three zones, where the STZ photomicrograph is taken under compressive loading, while the other two zones are unloaded.

Articular cartilage behaves like a sponge but allows fluid to escape only slowly. Typically, from the mechanical point of view, it can be considered biphasic with the solid phase modelled as an incompressible viscoelastic material reinforced by a collagen network and undergoing finite strain. The fluid phase is inviscid and incompressible. For rapid loading articular cartilage responds as a solid because there is no time for the fluid to escape from the tissue. Because of the high content of proteoglycans, the material is also negatively charged, which arises from the sulfate and carboxyl groups. Therefore, several modelling approaches consider a triphasic model to also capture the swelling of articular cartilage [38,39].

#### 2.4.1. Material models

**Figure 8.**
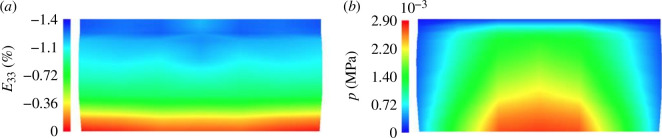
Numerical results of an unconfined compression test on a three-layered cube of cartilage obtained using the model described by Pierce *et al*. [41] without the viscous part. The left image shows the change in the Green–Lagrange strain component along the direction of loading for axial compression from 5% to 6% strain. The right image illustrates the fluid pressure distribution obtained from 1% axial compression after the initial 5% strain. Adapted from Pierce *et al*. [40].

Most material models of articular cartilage consider the material to be biphasic and fibre-reinforced based on poroelasticity or poroviscoelasticity, although many contributions adopt a linear model for the solid. However, it is important to consider the nonlinear response of the tissue, as is done in the present review. A prime modelling example describing the continuum basis and computational aspects with an application to articular cartilage is documented by Pierce *et al*. [40]. Figure 8 shows numerical results of an unconfined compression test of a three-layer thick cartilage cube. In particular, the left figure illustrates the distribution of the change in the 33-component of the Green–Lagrange strain tensor (along the loading direction) between 5% and 6% axial compression. The right figure illustrates the distribution of fluid pressure resulting from 1% axial compression after the initial 5% strain. An extension to the viscoelastic case is provided by Pierce *et al*. [41] in which the viscoelastic formulation was used as described in section 6.10 of Holzapfel [31]. In addition, by Pierce *et al*. [41], the permeability was considered to be dispersed.

**Figure 9.**
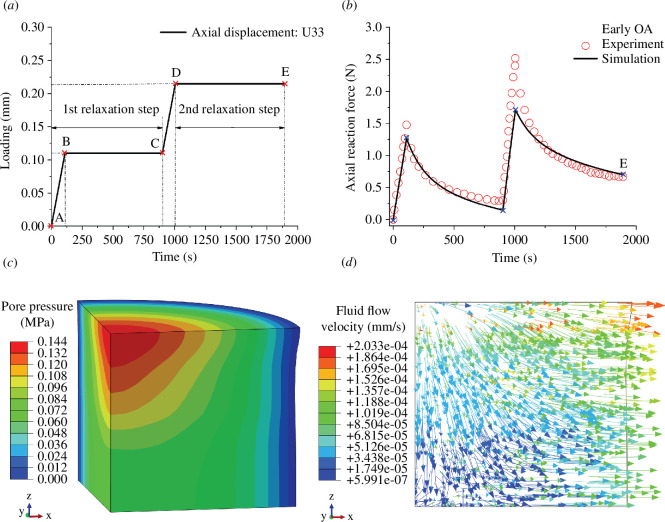
Unconfined compression test of a cylindrical sample of human articular cartilage with mild osteoarthritis. (*a*) Loading protocol for the finite element simulation of the test described by Julkunen *et al*. [42]. (*b*) Comparison of the relaxation data of the axial reaction force with results of the simulation based on the poroviscoelastic model documented by Liu *et al.* [43]. (*c*) Distribution of the pore pressure for a quarter of the cylinder at point D (figure 9*b*) indicating a high pressure in the centre region of the top specimen surface (red). (*d*) The arrows show the directions of the fluid flow indicating the drainage from the lateral surface. Adapted from Liu *et al*. [43].

Data from unconfined compression tests have been provided by Julkunen *et al*. [42] and these data have been used in the finite element simulation by Liu *et al*. [43]. Cylindrical samples from human articular cartilage were obtained for use in experimental tests; for details of the sample size and composition, see table 1 in Julkunen *et al*. [42]. Stress-relaxation tests for unconfined compression of several samples were performed in two steps, according to the loading protocol shown in figure 9*a*. The red circles in figure 9*b* correspond to the relaxation data of the axial reaction force obtained from sample II in Julkunen *et al*. [42]. This figure also compares the poroviscoelastic model in Liu *et al*. [43] (solid curve) with the data, which indicates a good agreement. Point A in figure 9*a*,*b* indicates the initial point of the first relaxation step. The distribution of the pore pressure shown in figure 9*c* corresponds to the maximum axial displacement, i.e. point D in figure 9*b*. The pore pressure on the cylindrical surface is maintained at zero during the test because of the free draining of the fluid allowed from this surface. The pore pressure is highest in the center region of the top surface, as shown in red. Due to this specific distribution of the pore pressure gradient, the fluid flows from higher to lower pressure regions until it extrudes through the lateral surface to freely drain, as shown in figure 9*d*.

Several review papers emphasizing different aspects of articular cartilage biomechanics are available. For example, Halloran *et al*. [44] describe multi-scale modelling and simulate the biomechanical function of cartilage. Multi-phase mechanical models of articular cartilage and their use towards an understanding of the connection between mechanical function, pathologies and possible interventions are reviewed by Klika *et al*. [45]. The article by Seyedpour *et al*. [46] summarizes various MRI applications used for assessing the stresses and material properties of cartilage based on mathematical modelling (e.g. [47,48]), and also addresses the limitations of different material models.

### 2.5. Skin

**Figure 10.**
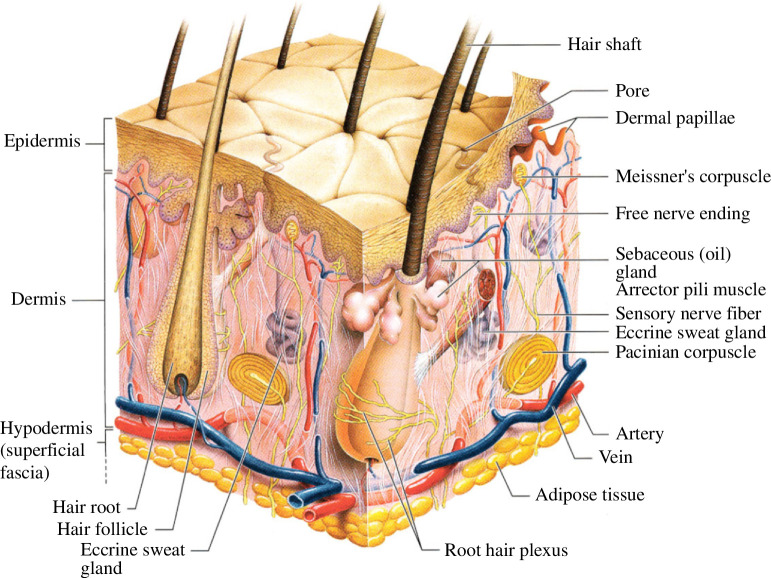
Multi-layer structure of the skin. Adopted from Marieb [50].

Human skin is the largest organ of the body in respect of surface area, and it accounts for about 15% of the body weight [49]. It protects us against external toxic agents and from traumatic injury to our internal tissues and organs as well as providing a means of temperature regulation. Skin consists of three main layers, namely the epidermis, dermis and hypodermis, each of which has a distinct structure, see figure 10. The epidermis is a waterproof layer which itself consists of several layers each having a different structure and function. It consists mainly of keratinocytes which synthesize the keratins, one of a family of structural fibrous proteins, that endow the epidermis with toughness. The outermost layer of the epidermis behaves as a stiff, isotropic sheet [51], and it is mainly responsible for the overall mechanical behaviour of the epidermis. Underneath is the dermis, which is a layer of connective tissue with a dense network of collagen that provides strength. The collagen fibres are closely connected to networks of elastic fibres leading to the typical fibre-reinforced composite. It is the primary load-bearing structural layer under tensile loads. Below the dermis is the hypodermis which consists mainly of connective and adipose tissues, which provide a cushioning effect. Pre-stretches and associated tensions are present in the skin and related to the collagen structure. The pre-stretches depend on age, location and disease, and they are identified by the Langer lines associated with the orientation of stretch [52,53]. For more details on the structure of the skin we refer to e.g. chapter 22 in Alberts *et al*. [54], Marieb [50], Kanitakis [49] and Limbert [55].

**Figure 11.**
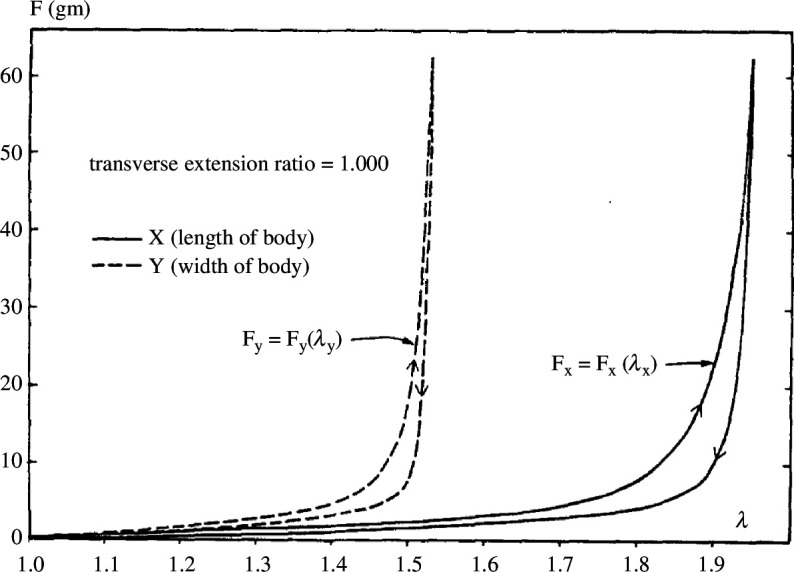
Force versus stretch curves of rabbit skin. The solid curves show the loading and unloading for fixed stretch $\lambda_{y}$, while the dashed curves present the loading and unloading for fixed stretch $\lambda_{x}$. A distinct level of anisotropy and nonlinearity can be seen. Adopted from Lanir & Fung [60].

Identification of dermal collagen in human skin is typically achieved using second harmonic generation imaging. Even *in vivo* depth scans are possible because the layers are very thin [56]. Typically, the three-dimensional structure of collagen can be used to determine the mean fibre orientation and the dispersion [57, 58]. The structure can also be investigated with the combination of histological images to determine the fibre dispersion [59]. Associated with the collagen fibres is the anisotropy in the material response and the nonlinear strain stiffening. Figure 11 shows a typical relationship of force versus stretch obtained from planar tension tests of rabbit skin with one stretch fixed [60].

In particular, the study by Annaidh *et al*. [61] investigates the mechanical behaviour of excised human skin under uniaxial tension tests along different directions with respect to the Langer lines (parallel, perpendicular or at $45^{\circ }$). The results suggest that there is a correlation between the Langer line orientations and the preferred orientation of collagen fibres. Corr *et al*. [62] developed a method for testing the tensile properties of skin in the axial and transverse directions and the changes in those properties after injury and subsequent scar formation. For a more recent review see Corr & Hart [63]. There are several other methods available for measuring the mechanical properties of skin such as biaxial tests [64] and bulge tests [65], which also show that skin is a viscoelastic and strain-rate dependent material [55].

#### 2.5.1. Constitutive modelling of the skin

**Figure 12.**
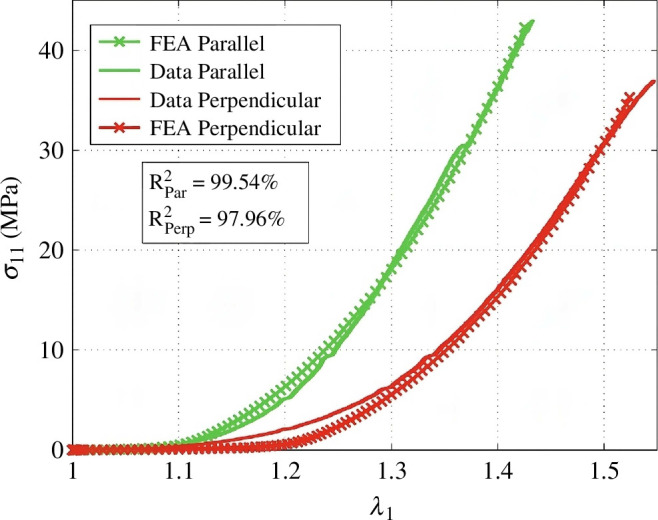
Comparison of data from a finite element analysis (FEA) with experimental data from uniaxial extension tests performed on human back skin. Parallel and perpendicular refer to the Langer lines. Adopted from Annaidh *et al*. [61].

Several constitutive approaches that have been used to model the mechanical properties of soft tissue, based on the theory of nonlinear elasticity or viscoelasticity, have been described by Limbert [55], not all of which have been used for skin. The angular integration approach of Lanir [66] was used to model the mechanical properties of skin [67,68]. The paper by Annaidh *et al*. [61] used the generalized structure tensor approach with axisymmetric distributed collagen fibre orientations to fit the data of uniaxial extension tests of human back skin [69]. The parameters were then used to perform a finite element simulation with ABAQUS [70] and the numerical results were in good agreement with data obtained from tissue samples parallel and perpendicular to the Langer lines (figure 12). The studies by Tonge *et al*. [71] and Tepole *et al*. [72] also use the generalized structure tensor approach [69] based on thin shell models.

### 2.6. Cornea

**Figure 13.**
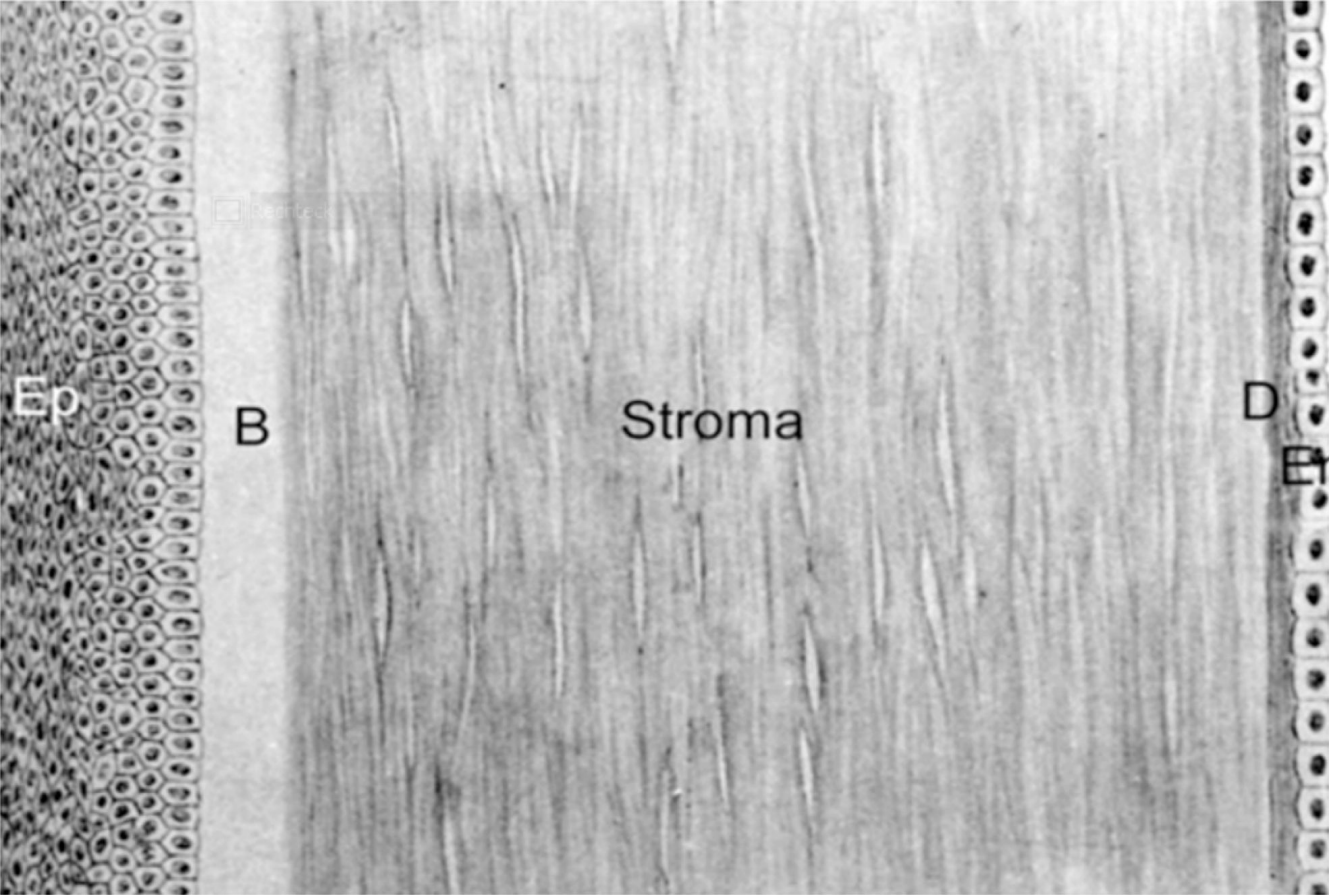
The five layers of the human cornea: epithelium (Ep), Bowman's layer (B), Stroma, descemet membrane (D) and endothelium (En). Adopted from Manganiello [74].

**Figure 14.**
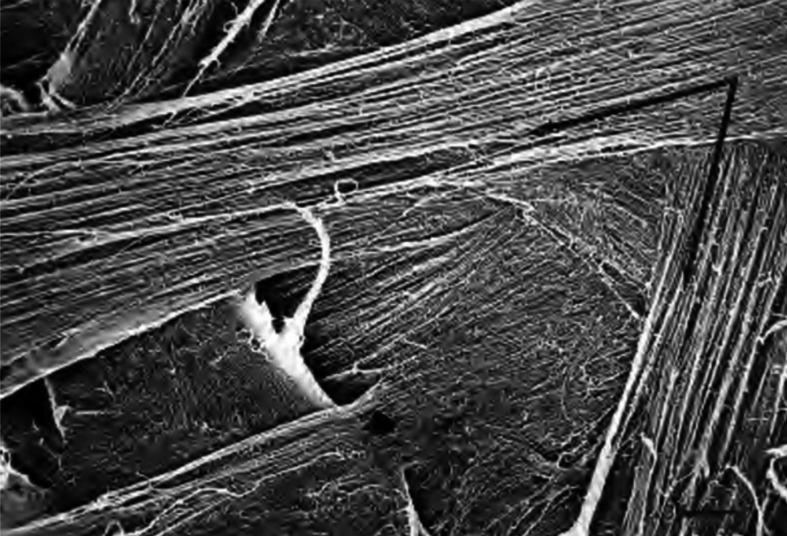
Collagen fibres in the corneal stroma along with sparsely populated keratocytes, about 200 lamellae with fibrils (5–6 μm thick). Adopted from Radner *et al*. [76].

The cornea is a convex dome-shaped shell with a non-uniform thickness (smaller at the center than at the extremities, where it is surrounded by the limbus). Geometrical parameters can be found in, e.g., Dubbelman *et al*. [73]. The cornea is the front part of the eye; it protects the internal eye components and contributes optically. It is a transparent composite material that refracts light, accounts for about two-thirds of the eye’s optical power (about 43 dioptres), and is responsible for focusing images onto the retina. Figure 13 shows a schematic of the cornea composition. The outermost layer is the corneal epithelium (Ep) made up of several layers of cells, and it serves as a barrier to protect the cornea. The Bowman’s layer (B) forms a membrane consisting of collagen fibrils with no clear architecture. The stroma is the middle layer, which provides 90% of the total thickness. It is essentially a composite material formed by collagen fibrils dispersed within a hydrated matrix that includes, in particular, proteoglycans, proteins and keratocytes. The collagen fibrils consist of collagen types I and V, and are the major constituents of lamellae (0.2–2.5 μm in thickness and 0.5–250 μm in width, see Komai & Ushiki [75]) in the stroma. Scanning electron microscope studies have shown that lamellae branch and interweave and that there is an in-plane angle between adjacent lamellae [76]. In the stroma, the collagen fibrils have preferred orientations along the superior–inferior (S–I) and nasal–temporal (N–T) directions, see, e.g., Daxer & Ratzl [77]. Related microscopic images show that the three-dimensional organization of the collagen lamellae is characterized by a greater extent of lamellar interlacing [76], and that collagen lamellae are inhomogeneous collagen structures along the anterior–posterior direction [78], see also figure 14. The remaining two layers are the Descemet’s membrane (D), comprising mainly of collagen fibres and the endothelium (En), which is a single layer of hexagonal shaped cells.

The mechanical properties of the cornea are governed by the complex collagen fibril architecture. With support of the stiff limbus tissue, the cornea retains its spherical shape under the forces to which it is subjected, in particular, the intraocular pressure, which is considered to have a mean value of 15 mmHg [79]. In addition, the specific structure of the anterior part of the stroma contributes to its stable equilibria [80]. It is the interaction of the collagen fibrils (having a high tensile stiffness) with other matrix constituents that leads to the anisotropic mechanical property of a single lamella. The study by Anderson *et al*. [81] used porcine corneal trephinates which were subjected to a gradually increasing posterior pressure up to more than 100 mmHg, which is far beyond the physiological intraocular pressure. The results show that there is a linear relationship between the applied pressure and the displacement up to approximately 30 mmHg. Above that pressure a rapid nonlinear stiffening was observed. The experimental study by Elsheikh *et al*. [82] indicates a stiffening effect with age in all loading cycles. The authors developed generic stress–strain equations for ages between 30 and 99 years on the basis of a strong statistical association between stiffness and age. Due to the large number of lamellae in the stroma, it is a highly heterogeneous material with varying degrees of anisotropy and with regions of isotropy [83,84].

#### 2.6.1. Mechanical modelling of the cornea

**Figure 15.**
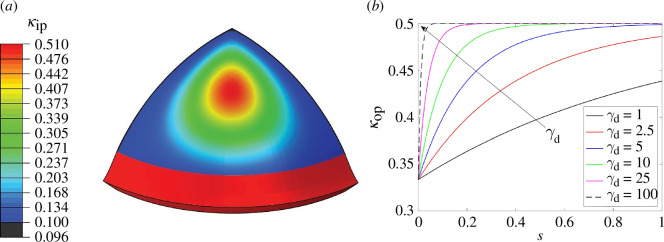
(*a*) Contour plot of the dispersion parameter $\kappa_{\mathrm{ip}}$ with respect to polar coordinates r, θ; (*b*) plots of the dispersion parameter $\kappa_{\mathrm{op}}$ through the cornea thickness from the anterior surface s=0 to the posterior surface s=1 depending on a nonlinearity parameter $\gamma_{d}$. Adopted from Wang & Hatami-Marbini [85].

Several constitutive modelling approaches have been developed for the description of the mechanical response of corneal tissue. The article by Wang & Hatami-Marbini [85] takes account of the dispersed collagen fibril structure using the non-symmetric dispersion model described by Holzapfel *et al*. [86]. Figure 15 illustrates the in-plane dispersion parameter $\kappa_{\mathrm{ip}}$ on a quarter of the cornea geometry, and additionally the distribution of the out-of-plane dispersion parameter $\kappa_{\mathrm{op}}$ over the corneal thickness s∈[0,1]. For details on the notation, see Holzapfel & Ogden [86]. In particular, $\kappa_{\mathrm{op}}$ ranges between 1/3 and 1/2 and the transition between these two values is governed by a nonlinearity parameter $\gamma_{d}$, while $\kappa_{\mathrm{ip}}$ varies between 0.1 and 0.5 with intermediate values depending on polar coordinates r and θ. The model provides a good agreement with experimental pressure–displacement curves and the stress profile across the corneal thickness.

**Figure 16.**
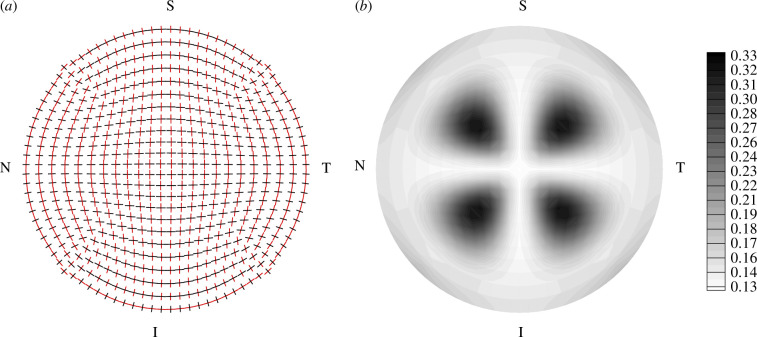
(*a*) Schematic of the orientation distribution of the two families of collagen fibrils in the outermost layer of the cornea (each pair of fibrils is visualized at the integration points of the finite elements); (*b*) distribution of the dispersion parameter κ, the same for each family of fibres. Adopted from Pandolfi & Holzapfel [84].

**Figure 17.**
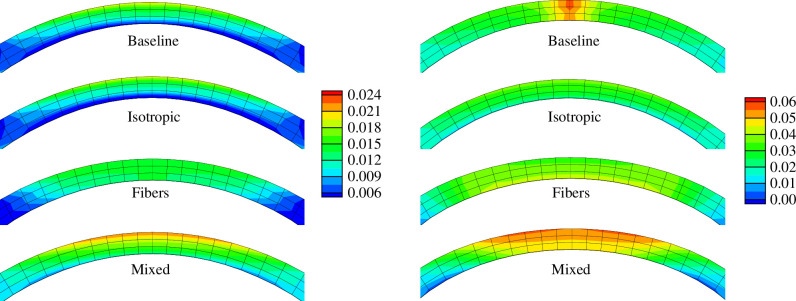
Distributions of the maximum principal Cauchy stress over the corneal thickness: (*a*) 16 mmHg; (*b*) 40 mmHg; baseline = dispersion parameters used in figure 16*b*; isotropic =κ=1/3 for both families; fibres =κ=0 for both families; mixed =κ=0 for the N–T direction and κ is according to figure 16*b* for the S-I direction. Adopted from Pandolfi & Holzapfel [84].

The computational model by Pandolfi & Holzapfel [84] used the constitutive model by Gasser *et al*. [87], the axisymmetric dispersion model and predicted well the experimental intraocular pressure–displacement data from Anderson *et al*. [81]. Thereby, for the first time, a realistic distribution of the fibre dispersion over the cornea was used; see figure 16. Figure 17 illustrates maps of the maximum principal Cauchy stress over the thickness of the cornea for two different levels of intraocular pressure and for different values of the dispersion parameter κ.

Another recent application of the symmetric fibre dispersion model was used to capture the mechanical response of the cornea [88]. It examined relative contributions of each part of the stroma to corneal mechanics and keratoconus pathogenesis (a disease of the eye that leads to progressive thinning of the cornea; for a recent review in regard to a biomechanical perspective, see Padmanabhan & Elsheikh [89]. The effect of local and global softening on the corneal vertical displacements was analysed. The study by Wang & Chester [90] developed a multi-physics model and robust numerical simulation capability by considering vitamin B2, ultraviolet light absorption and biomechanical changes caused by microstructural variations. Model calibration was performed by comparing the numerical results with a set of nanoindentation and inflation tests. The study by Pandolfi [91] used an approximation to the model by Gasser *et al*. [69], derived by Pandolfi & Vasta [92], to simulate the actual distribution of stress and strain before and after surgery, and also to simulate indentation tests.

Based on the angular integration approach, as outlined by Lanir [66], the paper by Pinsky *et al*. [93] proposes a mathematical model for a typical lamella including the strain energy of the collagen fibrils, obtained from X-ray data, matrix material and proteoglycan cross-linking. The proposed stromal model is based on an average of the lamella properties through the stromal thickness. The documented application is a prediction of astigmatism induced by a tunnel incision in the sclera, which was in agreement with published clinical data. The paper by Foong *et al*. [94] used a hyperelastic model including probability density functions for the percentage of fibres with a given orientation and crimp. This involved integration over an axisymmetric distribution of fibrils similar to that used in the angular integration approach. It was shown that the percentage of collagen fibres in the posterior equator was recruited faster as the intraocular pressure increased, with over 90% of the fibres recruited at an intraocular pressure with 15 mmHg, compared with approximately one third in the cornea and the peripapillary sclera.

Zhou *et al*. [95] map the collagen fibril magnitude and orientation on the three-dimensional geometry of the eye globe based on measurements of X-ray scattering. Finite element models with eye-specific geometry were used to obtain material parameters from experimental data from various donors. Material parameters for a constitutive model, the generalized structure tensor approach, as introduced by Holzapfel *et al*. [3], without a dispersion and two families of fibres, accounted for the collagen fibril density and orientation. The model was used in particular pressure–displacement curves for different regions of the ocular globe, and for different tissue ages. Very recently a fractional derivative approach was used to model the viscoelastic behaviour of human cornea [96]. Although the model is one-dimensional, a three-dimensional fractional derivative model such as developed by Nordsletten *et al*. [19] and Zhang *et al*. [20] has the potential to provide a more general framework to capture the time-dependent effects of the human cornea.

### 2.7. Intervertebral disc

**Figure 18.**
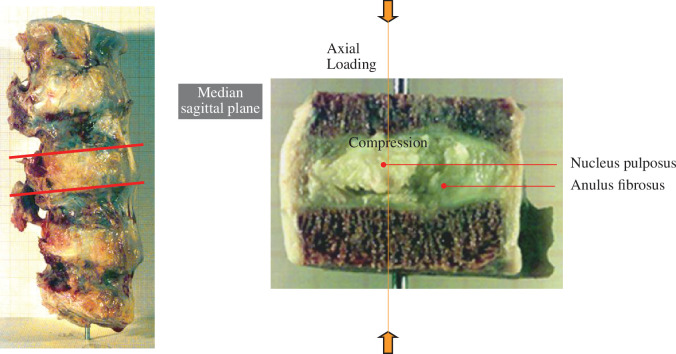
Part of a human spine showing several vertebrae and intervertebral discs. The zoom-up shows a single intervertebral disc with adjoining vertebrae, consisting of a gelatinous nucleus pulposus, the annulus fibrosus and cartilaginous endplates. Partly adopted from Holzapfel & Ogden [97].

**Figure 19.**
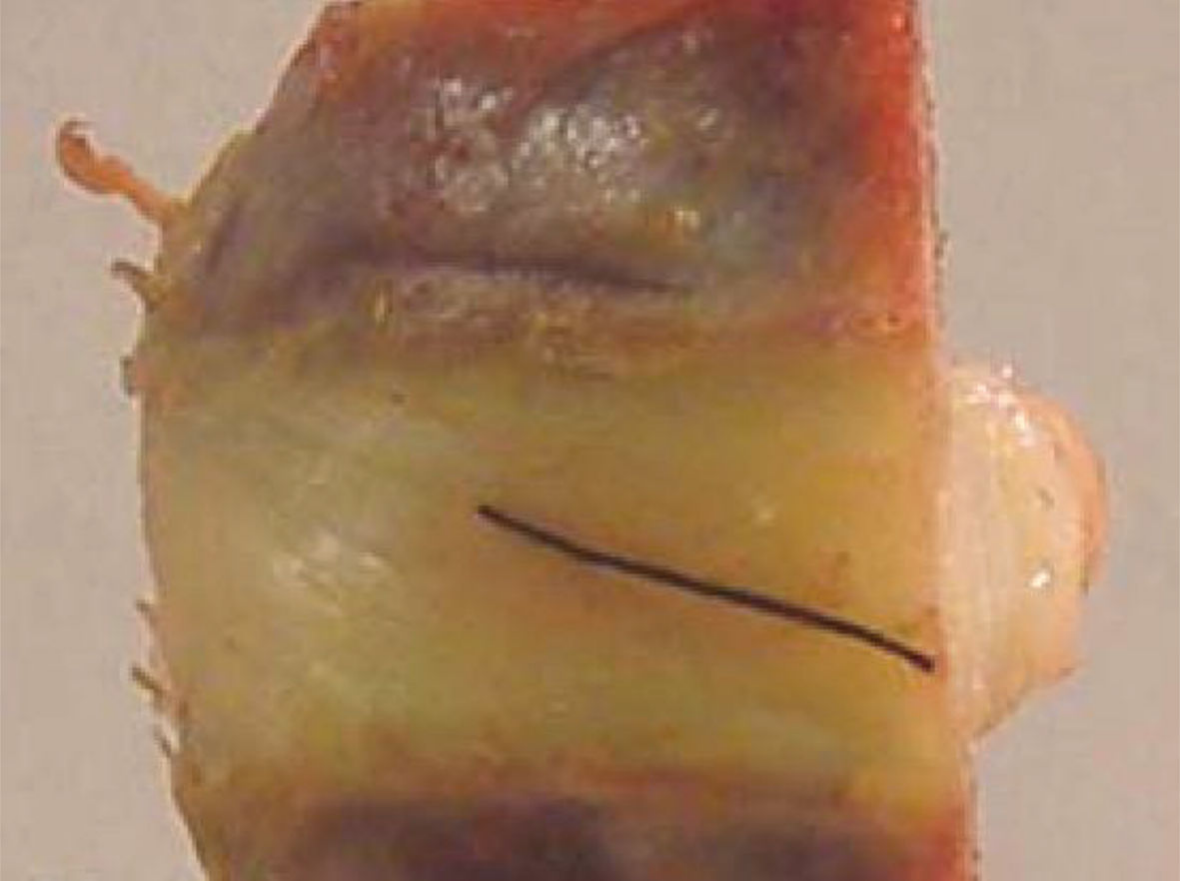
Front view of a hemidisc between two vertebrae: black thread identifies the orientation of collagen fibre bundles. Also seen is the swelling of the nucleus pulposus. Modified from Holzapfel *et al*. [99].

The intervertebral disc is located between two vertebrae and connected to thin layers of cartilaginous endplates, which have key roles in the distribution of load between the intervertebral disc and the vertebrae and in the diffusion of nutrients into the intervertebral disc. The central region of the disc is the gelatinous nucleus pulposus surrounded by the annulus fibrosus; see figure 18. The nucleus pulposus is a hydrated gel consisting of a large quantity of water and proteoglycans, which carry negatively charged proteins leading to the generation of osmotic pressure caused by the attraction of positive ions. The pressure in the nucleus pulposus is approximately 0.1–0.2 MPa at rest, while it may go up to 3 MPa during movement [98]. Figure 19 shows half of an intervertebral disc and adjacent vertebrae with swelling of the exposed nucleus pulposus illustrated.

The nucleus pulposus is permeable and supports shear and compression. The typical shear modulus of a healthy human nucleus pulposus is approximately 0.2 kPa, while the compression modulus is three orders of magnitude larger. Under dynamic loading the nucleus behaves like a viscoelastic solid. When the nucleus degenerates its mechanical properties change significantly. For example, the shear stiffness increases [100], while the swelling pressure decreases (e.g. [101]). For more details about the mechanical properties of the nucleus pulposus, see Elliott *et al*. [102].

**Figure 20.**
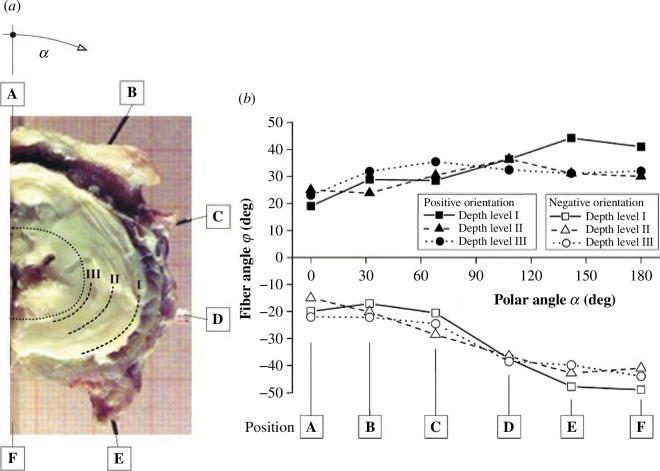
Distribution of the fibre angle φ at different locations around the half intervertebral disc: (*a*) representative depth levels I, II and III and locations A to F around the half intervertebral disc, and the polar angle α associated with the circumferential position; (*b*) variation of the fibre angle φ with the polar angle α along the locations A to F for two successive lamellae. Modified from Holzapfel *et al*. [99].

The annulus fibrosus is a stiff annular-shaped structure consisting of two symmetrically aligned families of collagenous lamellae that are embedded in an interstitial material that consists mainly of water, proteoglycans and non-collagenous proteins. The orientation angle φ of the collagen bundles varies with the polar angle α associated with the circumferential position according to |φ|=23.2+0.13α, see Holzapfel *et al*. [99]. On the other hand, the change in the radial direction is small. Figure 19 shows the angle φ of a collagen bundle, identified by a black thread. Figure 20*b* shows the regional variation of the orientation of two successive lamellae in one representative vertebra–hemidisc–vertebra unit. The angle φ of the collagen bundles is plotted against the polar angle α at three different depth levels (I, II, III) (α is zero for the midsagittal ventral position, see figure 20*a*). Levels I and III are near the outer and inner surfaces, respectively, and relate to single lamellar specimens, while level II is at an intermediate depth. Figure 20*a* shows half an intervertebral disc indicating the location of the depth levels and the polar angle α.

When the intervertebral disc is compressed radial forces are generated, which transmit to the annulus fibrosus. Hence, when the nucleus is under compression the lamellae provide the load-bearing capacity of the annulus fibrosus. Thus, the mechanical behaviour of the entire annulus fibrosus is governed by (i) the tensile properties of the collagen bundles within the lamellae and (ii) the regional variation of the orientations of the lamellae. In general, the annulus fibrosus is subjected to nonlinear compression, tension, bending and torsion, and its material properties are anisotropic, viscoelastic and inhomogeneous. The inhomogeneity is manifested by regional and radial variations.

#### 2.7.1. Mechanical modelling

**Figure 21.**
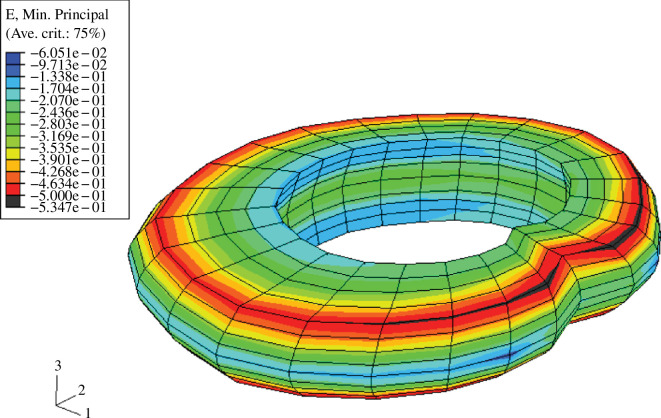
Distribution of maximum principal strains for the annulus fibrosus at 1.6 mm axial displacement, and geometry taken from a CT scan. Adopted from Eberlein *et al*. [103].

Here, we discuss the constitutive and computational modelling of the intervertebral disc and the spine, noting that the annulus fibrosus is clearly anisotropic. Several models have been developed based on the standard artery model by Holzapfel *et al*. [3], with the fibre part given by


(2.18) $$\begin{array}{lcr} & \Psi_{\mathrm{fib}}=\frac{k_{1}}{2k_{2}}\{\exp[k_{2}(I_{4}-1{)}^{2}]+\exp[k_{2}(I_{6}-1{)}^{2}]-2\}, & \end{array}$$

where $I_{4}$ and $I_{6}$ are the squares of the stretches in the two fibre directions. For the isotropic part, $\Psi_{\mathrm{iso}}$ the neo-Hookean model (2.7) is frequently used for the matrix material of the annulus fibrosus. The first application of this model to the intervertebral disc is documented by Eberlein *et al*. [103]. The constitutive model (2.18) was implemented in the finite element program ABAQUS [70] to analyse the deformation and strain behaviour under uniaxial compressive forces for a specific segment from a spine, the geometry of which was obtained from a CT scan. Figure 21 shows the distribution of the maximum principal strain values for the model with an axial displacement of 1.6 mm. The model is able to predict smooth and physiological strain distributions in the annulus fibrosus. The same model was used in a finite element study of, in particular, flexion–extension, lateral bending and axial torque of a human lumbar spine segment including the heterogeneity of the annulus fibrosus and its anisotropy [104]. There is a good correlation between these loading results and the experimental data for extension and axial torque.

Another study using the constitutive model (2.18), without reference to its source, is documented by Jacobs *et al*. [105], in which a compressible and isotropic model was used for the matrix of the annulus fibrosus. Finite element results were compared with experimental data from time-dependent confined compression tests documented by O’Connell *et al*. [106], thus indicating the need for considering multiple loading protocols. A slightly modified version of the constitutive model (2.18) was used by Tamoud *et al*. [107] to predict the anisotropic multi-axial damage response of the intervertebral disc up to failure of the fibres within the lamellae. The studies by Kandil *et al*. [108,109] use a quadratic form of $I_{4}-1$ for the fibre energy function (2.18). The paper by Caner *et al*. [110] uses a quartic form of $I_{4}-1$ for the energy stored in the collagen fibres, and the authors also include a fibre–matrix interaction term. It is a microplane model that involves an orientation distribution of the fibres, and provides descriptions of the anisotropy in the annulus fibrosus, but is computationally expensive. Another more recent microplane model for the annulus fibrosus based on the study by Caner *et al*. [110] was proposed by Ghezelbash *et al*. [111] using two families of fibres.

An invariant-based model with nine independent material constants for the annulus fibrosus was suggested by Klisch & Lotz [112]. This article displays model fits in comparison with experimental data, which are not in perfect agreement and thus suggests that the predictions of this particular model are limited. Moreover, the physical motivation for the choice of the models and invariants is unclear. An exponential form of the stretches in the direction of the two fibre families accounting separately for the toe region and the subsequent linear response was proposed by Zhou & Willing [113]. With different functions for the toe and linear regions; however, it may lead to issues within a three-dimensional finite element environment. The paper by Ehlers *et al*. [114] compares the predictions of a cylinder representing the nucleus pulposus under compression and torsion with experimental results from Iatridis *et al*. [115], and shows that the fit of the biphasic model used is satisfactory with respect to torsion.

### 2.8. Ligaments and tendons

Ligaments and tendons are connective tissues comprising a dense arrangement of aligned fibres. Ligaments consist of parallel tough collagen fibre bundles; they connect bone to bone and accommodate their relative motions while maintaining bone and joint integrity. On the other hand, tendons connect muscle and bone and transmit the forces generated by muscle contractions. Ligaments and tendons have a very similar structure. Like most soft tissues, their main constituent is water, consisting of 65–70% of the total weight. The other constituents are collagen, mainly type I, which makes up approximately 70–80% of the dry weight, elastin, which accounts for a much smaller percentage of the dry weight, and a small percentage of proteoglycans.

**Figure 22.**
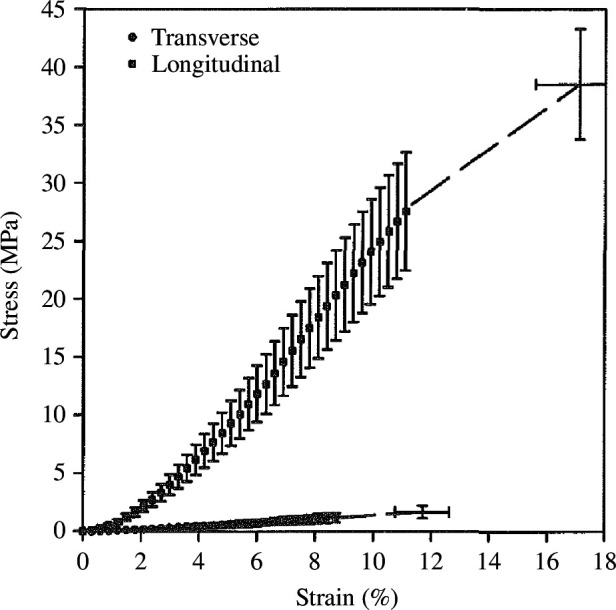
Nominal stress versus strain, (λ−1)100 (in %), for human medial collateral ligaments stretched along the longitudinal and transverse directions of the collagen fibres, averaged over nine (longitudinal) and seven (transverse) specimens. Adopted from Quapp & Weiss [116].

In terms of mechanical properties ligaments and tendons are rather stiff and strong along the fibre direction and weak orthogonal to it. Figure 22, shows the nominal stress versus strain for the average results of human medial collateral ligaments stretched along longitudinal and transverse directions relative to the collagen fibres [116]. From these data, one can deduce that the material is transversely isotropic. The general modelling framework for transversely isotropic elasticity was described by Holzapfel [31].

A frequently used strain–energy function, such as that documented in Weiss *et al*. [117], has the form


(2.19) $$\begin{array}{lcr} & \Psi =\Psi_{\mathrm{iso}}+\Psi_{\mathrm{fib}}, & \end{array}$$

where $\Psi_{\mathrm{iso}}$ is an isotropic strain–energy function often taken as the Mooney–Rivlin form [6,31], while $\Psi_{\mathrm{fib}}$ is often considered to have an exponential form such as that in (2.7)_1_. Based on the data displayed in figure 22, the paper by Weiss *et al*. [117] considered a tensile stress–stretch relation for ligaments and tendons composed of an exponential toe region followed by a linear region, and that the collagen fibres do not support compressive stresses. They proposed the model


(2.20) $$\begin{array}{lrlrr} & & & \frac{\partial \Psi_{\mathrm{fib}}}{\partial \lambda }=0,\;\lambda <1, & \end{array}$$

(2.21) $$\begin{array}{lrlrr} & & & \lambda \frac{\partial \Psi_{\mathrm{fib}}}{\partial \lambda }=C_{3}\{\exp[C_{4}(\lambda -1)]-1\},\;\lambda \leq \lambda^{⋆}, & \end{array}$$

(2.22) $$\begin{array}{lrlrr} & & & \lambda \frac{\partial \Psi_{\mathrm{fib}}}{\partial \lambda }=C_{5}\lambda +C_{6},\;\lambda \geq \lambda^{⋆}, & \end{array}$$

where $\lambda =I_{4}^{1/2}$ is the stretch along the collagen fibres, $\lambda^{⋆}$ is the stretch at the beginning of the linear region, and $C_{3},\ldots ,C_{6}$ are material constants, which are related by stress and stiffness continuity at $\lambda =\lambda^{⋆}$. The study by Shearer [118] uses a constitutive model of the form (2.19) with $\Psi_{\mathrm{iso}}$ having the neo-Hookean form of (2.7)_1_, $\Psi_{\mathrm{fib}}$ omitted for $I_{4}<1$ and for $I_{4}>1$ accounting for the fibril crimping with different forms for crimped and uncrimped fibrils. In a follow-up paper, the authors fitted the constitutive model to data from two different tendons up to strains of approximately 10% [119]. The energy functions for the collagen and the elastin by Henninger *et al*. [120] were based on the model (2.20)–(2.22) and the energy of the matrix was taken to have an isotropic form according to Veronda & Westmann [121].

**Figure 23.**
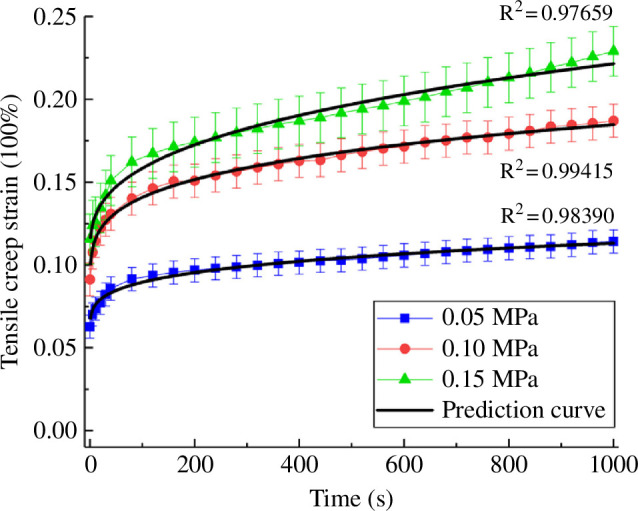
Creep tests for three different stress levels of a bovine periodontal ligament in comparison with a theoretical prediction using an isotropic energy function with a viscoelastic model. Adopted from Zhou *et al*. [123].

Ligaments are viscoelastic as shown, e.g. for a bovine periodontal ligament by Najafidoust *et al*. [122] and Zhou *et al*. [123]. Figure 23 provides an example of creep tests for three different stress levels showing a strong change in the first 200 s. The plots compare experimental data with model predictions based on the combination of an isotropic hyperelastic energy function with a viscoelastic model. Tendons are also viscoelastic, see e.g. the review article by Fang & Lake [124]. A model based on the invariants of C and its rate $\dot{\text{C}}$ for human knee ligaments and tendons was developed by Pioletti *et al*. [125], although transverse isotropy was not considered. In a specific example, the model was used to fit experimental uniaxial data at different stretch rates with a satisfactory agreement. In Woo *et al*. [126] there is some discussion of the nonlinear properties of ligaments and tendons in addition to the constitutive description for their time-dependent behaviour including the Pipkin–Rogers model [127]. For references to other models, see the review article by Fang & Lake [124].

An important consideration for ligaments and tendons is how damage affects their mechanical properties [128]. A continuum model for incorporating accumulated damage within a tendon constitutive formulation was developed by Allan *et al*. [129] based on (2.20)–(2.22) together with a damage variable for the damage contribution of the fibre as documented by Balzani *et al*. [130]. The developed model shows good agreement with experimental results in creep cyclic fatigue. The paper by Knapp & Williams [131] proposes a damage model of an anterior cruciate ligament by taking a strain energy of the form $\Psi_{\mathrm{iso}}+\Psi_{\mathrm{fib}}$, with $\Psi_{\mathrm{iso}}$ of the neo-Hookean form of (2.7)_1_ and $\Psi_{\mathrm{fib}}$ according to (2.7)_2_. The strain–energy function is multiplied by a decreasing damage function that depends on the maximum fibre stretch. A comparison of the numerical results based on ABAQUS [70] with experimental data shows a good agreement.

### 2.9. Adipose tissue

**Figure 24.**
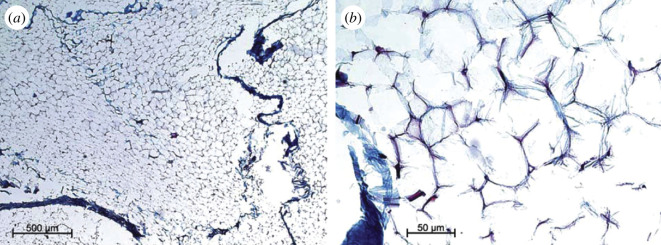
Histological images (chromotrope-aniline blue staining with a 5 μm thick section) illustrating the microstructure of human adipose tissue. Adipocytes are surrounded externally by a network of collagen seen as black curves: (a) 5× and (b) 40× original magnification. Adopted from Sommer *et al*. [133].

Adipose tissue is a loose, soft connective tissue that mainly consists of adipocytes. They form a thick layer just below the skin (subcutaneous adipose tissue) or within the abdomen or surrounding internal organs (visceral adipose tissue). The totality of the adipose tissue in the human body is referred to as an organ. Each location contains white adipose tissue, which is responsible for energy storage, and a much smaller amount of brown adipose tissue in adults, which provides protection against hypothermia. Adipocytes are lipid-filled cells with a phospholipid cell membrane and the external support of collagen-based connective tissue, the so-called reinforced basement membrane, with a thickness of around 100 nm and a sheet-like type IV collagen [132]. They are arranged as lobules within an open-cell foam-like structure through which are threaded the interlobular septa, which consist mainly of a collagen type I fibre network. For more details of the microstructure, see Comley & Fleck [132]. Figure 24 shows representative histological images, with chromotrope-aniline blue staining, indicating the microstructure of human adipose tissue. For a description of different types of adipose tissue, see Koenen *et al*. [134].

**Figure 25.**
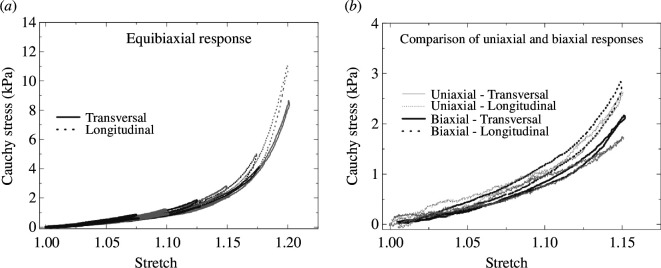
Examples of the Cauchy stress–stretch results from experiments on human abdominal adipose tissues for (*a*) stepwise equibiaxial tension (stretch increment is 0.025) and (*b*) uniaxial and biaxial tension tests with comparison for two orthogonal directions. Plots are shown after pre-conditioning. Adopted from Sommer *et al*. [133].

The mechanical properties of adipose tissue are important for understanding plastic surgery, cosmetics, repair of trauma or wound healing, for example. Abdominal adipose tissues can be characterized as nonlinear, anisotropic and viscoelastic biological materials [133]. A review of experimental tests for the characterization of the mechanical properties of subcutaneous adipose tissue is presented by Sun *et al*. [135]. Basically, uniaxial, biaxial, shear, unconfined compression and indentation tests are documented, sometimes in combination with creep and relaxation tests. For example, figure 25 illustrates equibiaxial stress–stretch responses of human adipose tissues in addition to plots showing a comparison of uniaxial and equibiaxial responses in two orthogonal directions under quasi-static testing. This sample suggests that the anisotropy is only minor. Figure 25*b* shows that the stresses are slightly smaller for the uniaxial data when compared with the biaxial data. In each case the nonlinear stiffening is clearly noticeable, while the dissipation is rather low, which, however, increases with the rate of loading.

**Figure 26.**
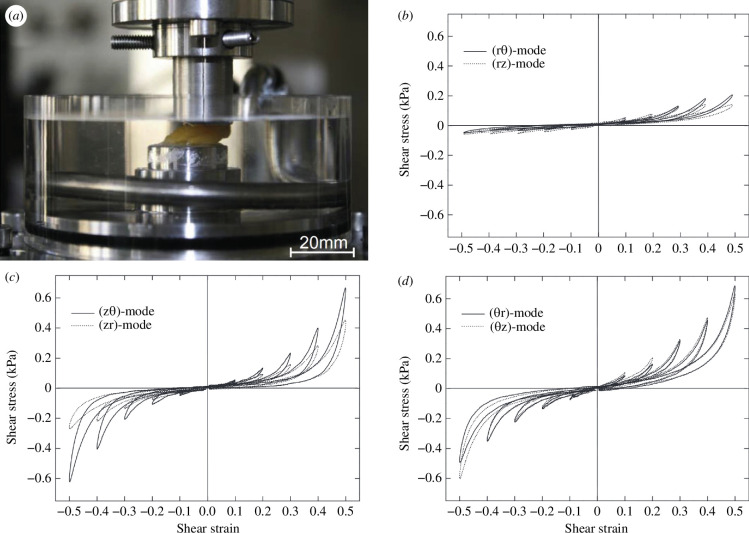
(*a*) Cube-shaped sample of adipose tissue was inserted into a triaxial testing device and subjected to simple shear loading. Plots of the shear stresses for different shear modes under cyclic shearing: (*b*) (rθ)-mode (solid curves), (rz)-mode (dotted curves); (*c*) (zθ)-mode (solid curves), (zr)-mode (dotted curves); (*d*) (θr)-mode (solid curves), (θz)-mode (dotted curves). The shear strain is increased in steps from 0.1 to 0.5. Adopted from Sommer *et al*. [133].

Adipose tissue also exhibits a highly nonlinear shear response which has not, as far as we know, been documented in the literature other than by Sommer *et al*. [133]; but see the more recent paper by Sun *et al*. [136], which follows the study [133] closely. In Sommer *et al*. [133], it was found that the shear response depends on the local adipose microstructure and/or orientation. Figure 26*a* shows a shear test conducted on a typical cube-shaped adipose tissue inserted into a triaxial testing device and subjected to simple shear loading. After seven sinusoidal simple shear cycles of pre-conditioning the final measurements of shear stress versus shear strain are documented in figure 26*b*–*d*. Therein the orientation in the body is labelled by θ (circumferential direction), z (vertically upwards) and r (radial). Figure 26 shows load cycles performed in different shear modes of a representative sample, with shear strain amplitudes increased in 0.1 steps. Strain softening can be seen in each cycle with increasing shear deformation. The hysteresis related to cyclic testing and the relaxation tests performed by Sommer *et al*. [133] indicate that adipose tissue has viscoelastic properties under shear. As can be seen from the plots, there is some degree of anisotropy because the response is softer in the (rθ) and the (rz) modes compared with the other ones. The degree of nonlinearity strongly depends on the plane in which the shear is applied.

**Figure 27.**
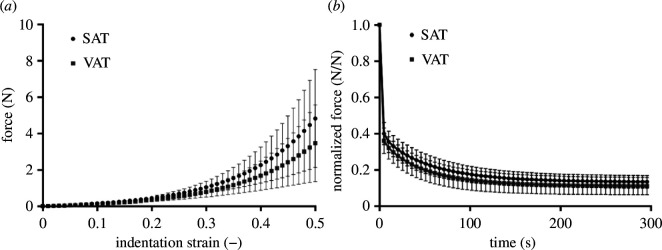
Curves of the mean data and related standard deviation from human abdominal adipose tissue using indentation tests: (*a*) equilibrium force-indentation versus strain plots; (*b*) normalized force versus time curves for subcutaneous adipose tissue (SAT) and visceral adipose tissue (VAT). Adopted from Fontanella *et al*. [137].

The study by Fontanella *et al*. [137] uses indentation tests with a spherical indenter (10 mm in diameter) to show that the mechanical properties of the subcutaneous and the visceral adipose tissue are different and time-dependent. Figure 27*a* quantifies the differences between the two types of adipose tissue in terms of the force and indentation strain relationship, while figure 27*b* shows the decay of the normalized force with respect to time indicating a rather strong early relaxation. Subcutaneous adipose tissue under blunt impact up to irreversible deformation is analysed by Lanzl *et al*. [138], which is relevant for the study of blunt force trauma as in e.g. car accidents.

For modelling the elastic nonlinear response of adipose tissue, the dispersion model from the study by Gasser *et al*. [69] is frequently employed, with the dispersion parameter κ used as a fitting parameter, while the matrix is modelled as a neo-Hookean material [133]. This constitutive approach has shown very good agreement with experimental data. As, in several cases, the elastic mechanical response is slightly anisotropic or even isotropic, several modelling approaches use the Ogden model [30]. The viscoelastic model as documented by Holzapfel [31] was used to fit relaxation data obtained from tests of samples of human abdominal adipose tissue [139]. For the elastic energy different isotropic functions were used and compared with very similar results. The study by Fontanella *et al*. [137] uses the Ogden model in addition to a viscoelastic term with different relaxation times to fit adipose tissue data from obese patients. The paper by Sun *et al*. [136] also used the Ogden model in combination with a one-dimensional quasi-linear viscoelastic model to obtain compressive and shear stress–strain data at different loading rates, and they fitted the model to experimental data from human abdominal subcutaneous adipose tissue.

### 2.10. Stomach

**Figure 28.**
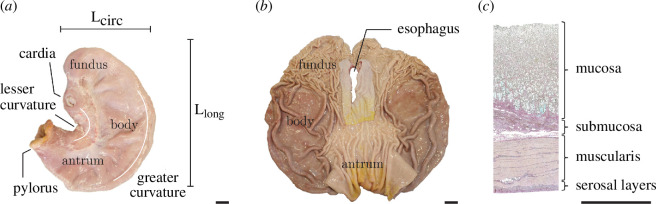
(*a*) Exterior view of an emptied, intact porcine stomach indicating its circumferential and longitudinal directions L_circ_ and L_long_, and its different regions, i.e. fundus, body and antrum; (*b*) interior view of the stomach opened up along the greater curvature; (*c*) illustrative histological slide of the cross-section of the stomach wall with four main layers: mucosa, submucosa, muscularis and serosa. Scale bars correspond to 2 cm in (*a*) and (*b*), and 2 mm in (*c*). Adopted from Holzer *et al*. [140].

The stomach is a bean-shaped muscular bag that sits in between the oesophagus and the intestine. It stores and processes the food which comes from the mouth through the oesophagus and enters the stomach. It consists of three regions, i.e. fundus, body and antrum (see figure 28*a,b*) with distinct histological properties. As in the biological soft tissues described in the previous sections, the stomach has a complex multi-layered wall with very distinct microstructures.

The stomach wall consists of four main layers, i.e. the mucosa, submucosa, muscularis and serosal layer [141], which have a total thickness of 3–4 mm. The mucosa, the innermost layer, exhibits distinct undulations called rugae, which unfold during food processing. It comprises multiple gastric glands, the lamina propria and the muscularis mucosae. The dimensions, distribution and orientation of the rugae depend strongly on location and are only a few millimetres in height. In particular, in the fundus they are tightly packed and dispersed. The submucosa is a thick and loose collagen network that contains, in particular, blood and lymphatic vessels, and facilitates the essentially independent motion of the muscular layer [142,143]. The muscular layer is formed of at least two interwoven strata which consist of muscle bundles surrounded by sheets of collagenous extracellular matrix. The outer stratum contains muscle fibres that are aligned axially. On the other hand, in the inner stratum the muscle fibres are mostly circumferential. Next to the muscularis is the serosal layer which includes a collagen network (also referred to as the subserosa) and a mesothelium, which serve to reduce friction in the abdominal cavity.

An analytic method for describing the three-dimensional stomach geometry was developed by Liao *et al*. [144], while a method that provides a full-field strain distribution of a three-dimensional gastric model between a reference configuration and a loaded configuration was proposed by Liao *et al*. [145]. A more recent study by Papenkort *et al*. [146] developed a three-dimensional geometry model of a real porcine stomach using photogrammetric reconstruction. More details on the anatomy of the stomach can be found in Lindberg & Lamps [141] and Mahadevan [147].

Several papers obtained data from uniaxial tests on stomach tissue. For example, on the basis of uniaxial tensile tests in different directions the studies by Zhao *et al*. [148], Jia *et al*. [149] and Carniel *et al*. [150] confirm that stomach tissue is anisotropic, nonlinear and viscoelastic. The passive elasticity of the wall is determined by the elastic fibres mainly in the submucosa and muscularis, while the smooth muscle fibres in the muscularis are responsible for the viscoelastic response [151]. The goal of the study by Friis *et al*. [152] was to compare elastic and viscoelastic mechanical properties of porcine and human stomach tissue from uniaxial tension and radial compression tests. It was found that there is a significant difference between the results for porcine and human stomach samples, and between the different parts of the stomach wall, the fundus, body and antrum, for both human and porcine tissue. In particular, circumferential samples from the antrum showed a higher stiffness and relaxation for human samples than for porcine samples. The results also suggest a difference in the results for anisotropy between the two species depending on the parts of the wall considered.

**Figure 29.**
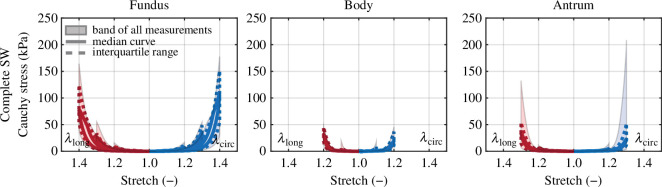
Equibiaxial stress–stretch results for consecutive stretches up to 1.1, 1.2, 1.3, and 1.4. The blue and red solid curves refer to the two directions of the extension. The dashed curves refer to the interquartile range of the data, while the shaded areas include all the measured data. Adopted from Holzer *et al*. [140].

However, uniaxial tests are insufficient to appropriately characterize the anisotropic mechanical properties [153]. At least planar biaxial tension tests are required supported by transverse compression tests and simple shear tests. Porcine stomach tissues exhibit an isotropic response at low stretches, while at higher stretches some anisotropy was evident [140]. Figure 29 shows Cauchy stress–stretch curves at the final cycle after pre-conditioning of equibiaxial extension in the complete stomach wall for each of the fundus, body and antrum.

Radial compression tests on porcine tissues show a rather weak and linear relationship between the first Piola–Kirchhoff stress and the stretch, down to a stretch of approximately 0.65, after which it increases exponentially. Simple shear tests show a linear behaviour up to 30% shear, which is then also followed by an exponential increase in the stress–strain curve. Both radial compression and simple shear tests of porcine tissues show a rapid stress relaxation [140]. In Aydin *et al*. [154] true stretch-controlled biaxial mechanical tests on porcine tissue show a degree of anisotropy in each of the three regions. Based on stretch-controlled biaxial tests, the study by Bauer *et al*. [155] also identified mild anisotropy with the highest stresses in the corpus in the longitudinal direction, followed by the antrum and the fundus. The maximum stresses in the circumferential direction were highest in the antrum, followed by the fundus and corpus. For a comprehensive review on the solid/fluid mechanics of the stomach including the analysis of chemical reactions, electrophysiology and electromechanics as well as mathematical and computational modelling of the stomach wall see Brandstaeter *et al*. [151].

A summary of some of the constitutive models that have been used is documented by Brandstaeter *et al*. [151]. Such models are typically of the decoupled form, as introduced in (2.19), where the isotropic part is frequently taken to be a neo-Hookean, Mooney–Rivlin or Ogden model and for the anisotropic part an exponential form of $I_{4}$ is often used, see (2.7)_2_. Sometimes the anisotropic part is considered to be a simple Fung-type model, see e.g. [154]. In the study by Klemm *et al*. [156], a novel three-dimensional electro-chemomechanical model of the gastric smooth muscle contraction was proposed. In particular, the mucosal layer was modelled on the basis of a neo-Hookean model and an anisotropic, nonlinear strain-energy function $\Psi_{\mathrm{fib}}$ according to


(2.23) $$\Psi_{fib}=\sum_{i=1}^{n}f_{i}\Psi_{fib\;i},$$

where n denotes the number of incorporated directions, and $f_{i}$ is a weight associated with the direction i such that $\sum_{i=1}^{n}f_{i}=1$, while $\Psi_{\mathrm{fib}\;i}$ denotes a strain–energy function of the form (2.7)_2_ for each i with compressed fibres are omitted. The muscular layer is modelled using a similar form for the passive response with the addition of a contribution for the active response, which depends on the level of chemical activation (see Gajendiran & Buist [157]), while a FitzHugh–Nagumo type model was adopted for inclusion of the electrophysiological response of the layer [158,159]. In the paper by Papenkort *et al*. [146] a finite element model was developed to simulate the pressure–volume response of a three-dimensional geometry model of a porcine stomach based on a one-term Ogden model in addition to an exponential function as in (2.7)_2_ but with $(I_{4}-1{)}^{2}$ term replaced by $(I_{4}-1{)}^{\beta }$, where β>2 is a material parameter obtained by fitting experimental data from Klemm *et al*. [156].

In Friis *et al*. [152] finite element calculations of the pressure–volume response for a complete stomach model comparing results before and after bariatric surgery are based on an isotropic stress response function combined with a viscoelastic stress term, as described in section 6.10 by Holzapfel [31]. A similar analysis was done by Fontanella *et al*. [160] using a different constitutive model for the elastic contribution, namely an exponential two-fibre family model associated with the longitudinal and circumferential directions.

## 3. Mechanobiology, mechanical homeostasis and modelling

### 3.1. Mechanobiology

Simply put, mechanobiology is the study of biological responses to mechanical stimuli. It has long been appreciated that mechanical loading affects the structure and function of tissues and organs—consider, e.g. Julius Wolff’s law of bone remodelling and Henry Gassett Davis’s law of soft tissue remodelling, both put forth in the nineteenth century. Yet, focused study of the underlying mechanobiological mechanisms began approximately 50 years ago and intensified largely over the past 25 years [161–168]. Many of the early modern studies assessed changes in gene expression or gene products when cultured cells were subjected to changing mechanical loads, particularly flow-induced wall shear stresses and cyclic in-plane stretching [169,170]. Our understanding of complex biological responses by cells to changes in their mechanical environment continues to increase rapidly with advances in technology for assessment, including atomic force microscopy, biaxial bioreactors, multi-photon imaging, optical tweezers, traction force microscopy and of course transcriptomics (bulk and single-cell RNA sequencing), proteomics and metabolomics. Importantly, the availability of genetically modified mice has also greatly aided the study of mechanobiology across many tissues and organs—one can selectively modify mechano-sensitive genes and evaluate the associated consequences in diverse tissues and organs.

We now know that many cell types are highly mechano-sensitive, including adipocytes within adipose tissue [171], cardiac myocytes within the heart [172], chondrocytes within cartilage [173], endothelial cells that line all blood vessels [174], fibroblasts within tissues ranging from skin to tendons [175], macrophages in many tissues [176], ocular cells within the eye [177], smooth muscle cells within airways, blood vessels and the bladder [178], and so forth. Consequently, mechanobiology is important for understanding development, homeostasis, disease and injury responses in myriad soft biological tissues and organs. Reviews of some tissue-specific impacts of mechanobiology on structure and function include those for blood vessels [179], brain [180], cartilage [181], heart [182], female reproductive organs [183], intervertebral disc [184], lung [185], skin [186], tendons [187] and valves within the heart [188]. Although many biological responses by cells are niche specific, that is specific to a particular tissue or organ, there are also many common processes that cells use to mechano-sense (assess) and mechano-regulate (assemble) their local environment, particularly the extracellular matrix [189]. Although often referred to simply as mechanotransduction, one can identify three primary cellular processes fundamental to mechanobiology: transduction (e.g converting information on the extracellular mechanical state into intracellular chemical or physical signals), transcription (i.e. mechano-sensitive changes in gene expression) and translation (i.e. conversion of transcriptional changes into gene products).

Of particular importance, cells often assess their local mechanical state via transmembrane protein complexes called integrins [190]. Composed of so-called α and β subunits, these dimeric complexes provide a mechanical linkage between the extracellular matrix and cytoskeleton, often actin filaments. Example integrins include α2β1 (with a high affinity for the extracellular matrix protein collagen) and α5β1 (with a high affinity for the extracellular matrix glycoprotein fibronectin). Collagen tends to endow tissues with stiffness and strength whereas fibronectin serves both to promote cell adhesion and fibrillogenesis of other extracellular matrix constituents. In addition to these two we also mention elastin. This extracellular matrix protein combines with elastin-associated glycoproteins such as the fibulins and fibrillins to form elastic fibres, which tend to endow tissues with compliance and resilience. Tissues that continually undergo large cyclic deformations, such as proximal arteries and the lung, contain abundant elastin; by contrast, tissues that primarily provide stiffness and strength, such as tendons (connecting muscle to bone) and ligaments (connecting bone to bone), consist mainly of highly oriented fibrillar collagens, noting that collagens are the most common proteins in mammals. Other key extracellular matrix components include the many different glycosaminoglycans and proteoglycans (i.e. glycosaminoglycans attached to a protein core). They play many roles within the extracellular matrix, including mechanical and mechanobiological [191,192]. Sensing of the extracellular matrix via integrins allows the cells to assess both the composition (ligand-dependent) and mechanical state (ligand-independent) of their extracellular environment—e.g. a cell can discern the difference between a substrate consisting of fibronectin versus collagen even if the stiffness is similar. It is becoming increasingly clear that mathematically modelling mechanobiology must account for both types of sensing. A particularly interesting example stems from a chimeric mouse model developed by Schwartz whereby the cytoplasmic domain of the α5 integrin subunit was replaced biologically with the cytoplasmic domain of the α2 integrin subunit. Hence, the cells still bind fibronectin (which in excess can be pro-inflammatory) via the extracellular domain of α5 but they signal via α2 as if they had bound collagen. When the α5/α2 chimeric mouse was crossed with a mouse model of Marfan syndrome characterized by reduced fibrillin-1 and increased fibronectin, the degree of aortic disease decreased [193].

In addition to sensing, cells also use integrins and cytoskeletal contractility to mechano-regulate (assemble) the extracellular matrix. That is, as described early on by Alberts *et al*. [54], cells appear to work on or fashion newly deposited extracellular matrix, presumably to endow the tissue with favourable mechanical properties such as anisotropy and compliance. As an example, vascular smooth muscle cells can synthesize and secrete collagens in the absence of integrins, but they cannot organize the secreted collagen without integrins and cytoskeletal contractility [194]; similarly, fibroblasts can only remodel extant collagen if they have intact actin filaments [195]. Among other mechano-regulated processes, it appears that cells must induce a deposition (or pre-) stretch in newly deposited collagen fibres to ensure stable remodelling of an artery [196]. Such a pre-stretch not only contributes to tissue homeostasis, for at least certain structural proteins it also contributes to proteostasis. For example, an optimally pre-stretched collagen fibre has increased resistance to proteolytic degradation [197], which increases its half-life. Interestingly, mechano-regulation of the extracellular matrix can also affect the local behaviour of other cell types. It has been shown e.g. that fibroblasts can direct macrophage migration simply by mechanically remodelling the matrix and that this far-field effect can exceed distances known for chemoattractant signalling [198].

### 3.2. Mechanical homeostasis

Fundamental to the study of mechanobiology is the concept of homeostasis, which was introduced in the 1920s by the American physiologist W. Cannon to describe *'How the human body reacts to disturbance and danger and maintains the stability essential to life'*. In brief, homeostasis is a biological or physiological process by which certain regulated quantities are maintained, within a tolerance, near a target value, often called a set-point. Common examples include core body temperature and interstitial fluid pH. It has long been thought that mechanical stress is one such regulated quantity, which for blood vessels dates back to pioneering work by Wolinsky & Glagov [199]. In particular, noting that the mean circumferential Cauchy stress in a pressurized tube is $\sigma_{\theta }=Pa/h$, where P is the distending pressure, a inner radius and h wall thickness, these investigators reported that the ratio a/h is nearly the same across mammals from mice to humans (which also have similar blood pressures), thus suggesting that intramural cells maintain wall stress or wall tension near favourable target values. Although it is unlikely that cells directly sense the continuum metrics of stress, strain or stiffness, these conveniently calculated quantities are expected to continue to serve as excellent correlates for analytical and computational models of mechanobiology [200].

**Figure 30.**
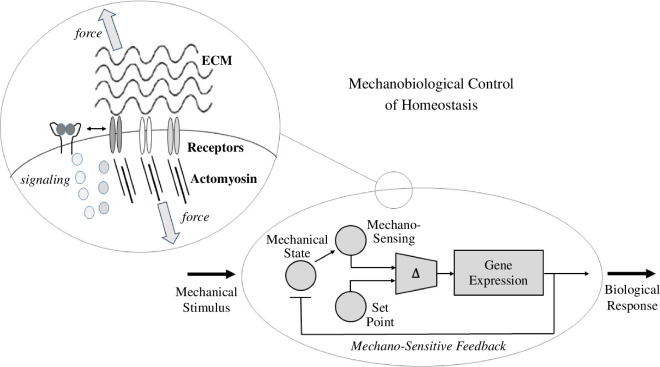
Schema of mechanobiological control of soft tissue homeostasis. Mechanobiology is defined as the study of biological responses to mechanical stimuli, illustrated here for a mechano-sensitive cell. From a mechanics perspective, one can imagine that the cell senses certain characteristics that define the mechanical state (e.g. stiffness) and these values are compared with target (set-point) values. If the deviation Δ between the sensed and the target value is within a particular tolerance, there is no change in gene expression. If the deviation Δ exceeds this tolerance, the response is often a change in gene expression leading to a change in gene products that can result in changes in geometry, composition and material properties. Homeostatic responses are defined by negative feedback designed to drive the mechanical state back toward normal. Finally, shown too (upper left) are components along the mechanotransduction axis: extracellular matrix (ECM), transmembrane protein complexes including integrins and mechano-sensitive growth factor receptors and cytoskeletal constituents including actin and myosin. Diverse second messengers serve to signal the mechano-stimulated cell to change gene expression.

There are two implicit aspects of mechanical homeostasis that are particularly important. First, homeostasis requires negative feedback to return a regulated quantity towards normal following a perturbation. Second, homeostasis is necessarily a stable process (see figure 30 for a schema of mechanobiological control of soft tissue homeostasis). As a result, compromised or lost mechanical homeostasis is often characterized by positive feedback that leads to an unstable process [201]. The concept of homeostasis has become central to computational modelling of cell-level, tissue-level and organ-level responses to changes in mechanical loading but there remains a need for continued research into fundamental mathematical concepts for describing and understanding mechanical homeostasis. One such concept is that of mechanobiological stability, which was introduced a decade ago and which incorporates concepts of control theory and dynamical systems to understand mechanisms and manifestations of mechanical homeostasis and its loss [202,203]. See, too, additional studies of mechanobiological stability ranging from cells to tissues [204–208].

### 3.3. Modelling in mechanobiology

Two common consequences of mechanobiological processes are stimulated changes in the mass (growth) and microstructure (remodelling) of a cell, tissue or organ. Although the importance of tissue growth has long been appreciated, mathematical modelling has been much more recent, as e.g. represented by establishment of the journal *Biomechanics and Modeling in Mechanobiology* in 2002. A few textbooks are now available [161,209,210] as are multiple review articles [211–213], among others. Notwithstanding the complexity of growth and remodelling (G&R) of soft biological tissues, there have emerged two primary approaches to mathematical modelling—the so-called theories of kinematic growth and constrained mixtures. In kinematic growth, first advocated in 1981 [214], and formalized more than a decade thereafter [215], one assumes that the total deformation gradient F can be decomposed via a multiplicative decomposition into a growth part and an elastic part, namely, $\text{F}={\text{F}}_{e}{\text{F}}_{g}$. It is further assumed that the stress-response, often inferred from a standard stored-energy function, depends solely on the elastic part of the deformation while growth is prescribed by a rate of change of the growth tensor, $\begin{array}{l} {\dot{\text{F}}}_{g} \end{array}$. Kinematic descriptions of growth include isotropic and transversely isotropic forms, namely,


(3.1) $${\text{F}}_{g}=\vartheta^{1/3}\text{I},{\;{\text{F}}_{g}=\text{I}+(\vartheta -1)\text{n}}_{0}⊗{\text{n}}_{0},$$

where ϑ is a growth parameter and the unit vector ${\text{n}}_{0}$ denotes a material direction in a reference configuration. Multiple forms for the growth tensor and its dependence on perturbations in chemomechanical states are discussed in Kuhl [216]. Therein it is noted that such growth can be prescribed in terms of factors that include mechanical stress and strain, nutrients, growth factors and hormones.

Alternatively, the constrained mixture theory was first introduced in 2002 [217]. Motivated by the desire to account explicitly for the different material properties, different natural configurations and different rates of turnover of the different structurally significant constituents that define cells, tissues and organs, in this approach one assumes individual rates of production and removal (or survival) of the different constituents, which are yet constrained to move together with the overall mixture. The equations of motion thus include individual mass balance equations (accounting for production and removal using standard continuum mixture relations) for each constituent but a single linear momentum balance relation that is informed by a rule-of-mixtures relation for the overall stored-energy function. Similar to the theory of kinematic growth, the constituent-specific deformation gradients admit a multiplicative decomposition: ${\text{F}}_{n(\tau )}^{\alpha }(s)={\text{F}}_{\tau }(s){\text{G}}^{\alpha }(\tau )$, where ${\text{F}}_{n(\tau )}^{\alpha }(s)$ denotes the deformation experienced by constituent α at current G&R time s relative to its individual natural configuration n(τ), ${\text{F}}_{\tau }(s)=\text{F}(s){\text{F}}^{-1}(\tau )$ denotes mixture-level deformations between G&R times τ and s, and ${\text{G}}^{\alpha }(\tau )$ denotes a cell-mediated deposition (or pre-) stretch induced in constituent α when it is deposited within extant matrix at G&R time τ∈[0,s]. Recall from above that cells use actomyosin activity to mechano-regulate newly deposited matrix, including orientation and pre-stretch. Whereas tissue-level deformations are potentially measurable, the deposition stretch must be prescribed constitutively (similar to an internal variable). In contrast to the kinematic growth approach, it is the rates of mass production (cell division, matrix deposition) and removal (cell death, matrix degradation), not growth deformations, that explicitly depend on differences in mechanical quantities from their set-point values; e.g. certain aspects of homeostasis can be modelled by requiring Δσ→0, where Δσ represents a difference between the current value of stress and its set-point. For an artery in maturity e.g. one often assumes homeostatic values for pressure-induced intramural stress and flow-induced wall shear stress. Regardless, it can be shown that mechanical homeostasis requires that rates of constituent production and removal must balance for each constituent, often written simply as $m_{o}^{\alpha }=\rho_{o}^{\alpha }k_{o}^{\alpha }$ where $m_{o}^{\alpha }$ is a basal rate of mass density production, $\rho_{o}^{\alpha }$ is the basal apparent mass density and $k_{o}^{\alpha }$ is the basal rate parameter associated with the removal (often via its constituent-specific half-life assuming a first-order kinetic decay). In contrast to the theory of kinematic growth, which typically employs standard stored-energy functions W for the elastic stress, the constrained mixture model requires a generalization of the stored energy, often using a rule-of-mixtures relation $W=\sum W^{\alpha }$ where $W^{\alpha }$ represents the energy stored per unit reference volume in individual constituents. As an example, it is common to let


(3.2) $$\rho (s)W^{\alpha }(s)=\rho^{\alpha }(0)Q^{\alpha }(s){\tilde{W}}^{\alpha }\left({\text{C}}_{n(0)}^{\alpha }(s)\right)+\int_{0}^{s}m^{\alpha }(\tau )q^{\alpha }(s,\tau ){\tilde{W}}^{\alpha }\left({\text{C}}_{n(\tau )}^{\alpha }(s)\right)d\tau ,$$

where ρ is the current mass density, $\rho^{\alpha }$ is the constituent-specific apparent mass density (thus $\rho^{\alpha }/\rho$ is a mass fraction), $Q^{\alpha }\in \left[0,1\right]$ is a constituent-specific survival function, ${\tilde{W}}^{\alpha }$ is a constitutive-specific stored-energy function that depends on its individual deformation (with ${\text{C}}_{n(\tau )}^{\alpha }(s)=({\text{F}}_{n(\tau )}^{\alpha }(s){)}^{T}{\text{F}}_{n(\tau )}^{\alpha }(s)$ the right Cauchy–Green tensor), $m^{\alpha }>0$ is a constituent-specific mass density production function, and $q^{\alpha }\in \left[0,1\right]$ is a constituent-specific survival function (capturing that fraction of a constituent that was deposited at past time τ that yet remains at current time s). It is the production and survival functions that depend on differences in mechanical quantities from homeostatic targets, with homeostasis requiring $m^{\alpha }\to m_{o}^{\alpha }$ and the rate parameter $k^{\alpha }\to k_{o}^{\alpha }$ (in the survival function). An early implementation of the full constrained mixture model focused on the progressive enlargement of intracranial saccular aneurysms [218], hence illustrating that G&R simulations can be used both to describe physiological adaptations and disease progression. Importantly, tissue-level growth and remodelling thus emerge naturally in the constrained mixture theory based on cell-level synthesis, deposition and degradation of different structurally significant constituents, as e.g. elastic fibres, fibrillar collagens, proteoglycans and contractile muscle. Given that cells comprise many different constituents (including microfilaments, intermediate filaments and microtubules), the constrained mixture approach can similarly be applied to an individual cell [219].

Additional illustrative approaches to modelling can be found in Goriely [209] and Taber [210] for the kinematic growth theory, and Nims and Ateshian [220] and Humphrey [221] for applications of the constrained mixture theory. See, too, the three aforementioned reviews [211–213]. One of the advantages of the kinematic growth model is its mathematical simplicity; one of the disadvantages of the constrained mixture theory is its computational expense. A recent report provides guidance, however, on implementing constrained mixture models for G&R using finite element methods [222]. Others have sought reduced constrained mixture approaches, including temporally homogenized [223] or mechanobiologically equilibrated [224], both of which reduce computational expense. Others extended the constrained mixture model to include reactive constituents [225].

Whereas early applications of both the kinematic and constrained mixture models focused on blood vessels [226,227,218], applications now span many different soft tissues. There have been many studies of G&R of the heart [228], including use of a kinematic growth model to study cardiac remodelling in exercise or disease [229], a homogenized constrained mixture model to gain insight into the possible reversible cardiac remodelling in modest hypertension [207], and a Lagrangian-based constrained mixture model of pathological cardiac remodelling [230]. A kinematic growth model of the brain predicted morphological changes due to differential mechanics of the inner core and outer cortex [231]. Mixture-based growth models for cartilage helped to define appropriate experiments [232], as any theory should, as well as to increase understanding of osteoarthritis [233]. A kinematic growth model helped to understand skin expansion as needed in many clinical settings [186]. Growth and remodelling models can also be used to understand native tissue structure and function, as e.g. in the eye [234].

### 3.4. Recent advances in mechanobiological modelling

Many exciting methods and applications continue to emerge in modelling mechanobiology. For cardiovascular applications, there is strong motivation to couple detailed mechanical calculations of the haemodynamics (e.g. via fluid–solid interaction, or FSI, simulations) with G&R simulations. Such approaches have been referred to as fluid–solid-growth simulations [235] and examples include study of the progressive enlargement of abdominal aortic aneurysms [236,237] and pulmonary artery maladaptation [238]. A recent general implementation within the SimVascular computational environment promises to increase capabilities further [239]. In addition, there is an increasing desire to relate specific changes in cell signalling with changes in cell behaviour or tissue- and organ-level G&R remodelling. This can be accomplished by coupling across scales [240]. Encouraged by past multi-modality coupling of agent-based and constrained mixture models [241], recent advances include coupling logic-based cell signalling models with agent-based models of cell responses [242] or coupling cell signalling models with tissue-level G&R models [243]. For example, one can use outputs from a constrained mixture model on changes in stress to drive changes in cell signalling via appropriate pathways, including altered integrin signalling or signalling through stress-sensitive receptors such as type I angiotensin-II receptors or transforming growth factor receptors. Such models promise to provide increased insight into natural biological processes, including mechanical homeostasis [244], but especially into possible therapeutic strategies. That is, whereas phenomenological (stress–strain or G&R) models cannot provide any insight into possible advantages of blocking different receptors or downstream targets (e.g. particular kinases), coupled cell signalling–constrained mixture models have promise for addressing such questions.

In summary, modelling in mechanobiology, particularly long-term growth and remodelling, has progressed significantly across multiple approaches for diverse tissues and there is considerable promise for such modelling to provide increased insight into normal and disease processes and their treatment.

## 4. Conclusions and future directions

As illustrated herein, soft biological tissues exhibit a tremendous niche-specific diversity consistent with different functions necessitating different microstructures. Mechano-sensitive cells establish, maintain, remodel and repair their extracellular matrix to endow each tissue with appropriate compliance, resilience, stiffness and strength, with most tissues exhibiting anisotropy, heterogeneity and under certain conditions viscoelasticity. Although the myriad matrix constituents within a given tissue, which can be of the order of 100 [245], each contributing significantly to both form and function, the primary mechanical function is typically captured at the tissue-level by simplified constitutive relations that consider elastic fibres, fibrillar collagens, aggregating proteoglycans and muscle and their general orientations. Although no truly microstructural model yet exists (that accounts for the many essential accessory proteins, glycoproteins and glycosaminoglycans as well as the effects of physical entanglements and different types of cross-links, both enzymatic and non-enzymatic), structurally motivated relations have yet to prove effective in capturing experimentally measured mechanical behaviours (in tension, compression and shear, as appropriate) in health and in many cases of disease. This review focused on a subset of such relations for illustrative purposes.

Given page-limits, however, this review was necessarily limited in scope. Among the different areas of importance not discussed, we suggest a need for increased attention to coupled roles of mechanics and modelling mechanobiology [213,210], biothermomechanics [246,247], cancer [248,249], development [250,251], immuno-mechanics [252,253], regenerative medicine [254,255] and wound healing and injury repair [256,257]. Toward this end, there is a need for a particular focus on modelling multi-scale mechanobiology [258,259]. Notwithstanding the advantages of *in vivo* animal models, particularly genetically modified mice, sophisticated *ex vivo* full-field methods for biomechanical assessment of complex nonlinear, anisotropic and heterogeneous properties should be advanced [260,261], and so too methods for multi-axial study of tissue equivalents [262,263], microfluidics [264] and organoids [265] should be pursued further with associated modelling and more attention to formulating and solving the associated initial-boundary value problems to better define the precise mechanical environment experienced by the cells.

Much has been accomplished in modelling soft biological behaviours, yet there remains a pressing need to move more and more towards to the goal of predictive spatio-temporal simulations. We must better predict normal tissue development, adaptations to altered loading, disease progression, responses to pharmacotherapy, responses to implanted biomaterials, responses to injury and prescribed physical therapies, responses to surgery and so forth. That is, much of our modelling must build on the many available constitutive descriptors of tissue properties at single times but extend them to account for cell-mediated temporal changes resulting from changes in gene expression. Only in this way, we will truly be able to impact medical device design and regenerative medicine and similarly the design of pharmacotherapies, physical therapy, surgical interventions and promises of future medicine, including novel transplant technologies and gene editing.

## Data Availability

This article has no additional data.
